# Infrapatellar Fat Pad/Synovium Complex in Early-Stage Knee Osteoarthritis: Potential New Target and Source of Therapeutic Mesenchymal Stem/Stromal Cells

**DOI:** 10.3389/fbioe.2020.00860

**Published:** 2020-07-28

**Authors:** Dylan N. Greif, Dimitrios Kouroupis, Christopher J. Murdock, Anthony J. Griswold, Lee D. Kaplan, Thomas M. Best, Diego Correa

**Affiliations:** ^1^Department of Orthopedics, UHealth Sports Medicine Institute, Miller School of Medicine, University of Miami, Miami, FL, United States; ^2^John P. Hussman Institute for Human Genomics, Miller School of Medicine, University of Miami, Miami, FL, United States; ^3^Diabetes Research Institute and Cell Transplant Center, Miller School of Medicine, University of Miami, Miami, FL, United States

**Keywords:** infrapatellar fat pad, synovium, mesenchymal stem/stromal cells, macrophages, osteoarthritis

## Abstract

The infrapatellar fat pad (IFP) has until recently been viewed as a densely vascular and innervated intracapsular/extrasynovial tissue with biomechanical roles in the anterior compartment of the knee. Over the last decade, secondary to the proposition that the IFP and synovium function as a single unit, its recognized tight molecular crosstalk with emerging roles in the pathophysiology of joint disease, and the characterization of immune-related resident cells with varying phenotypes (e.g., pro and anti-inflammatory macrophages), this structural complex has gained increasing attention as a potential therapeutic target in patients with various knee pathologies including osteoarthritis (KOA). Furthermore, the description of the presence of mesenchymal stem/stromal cells (MSC) as perivascular cells within the IFP (IFP-MSC), exhibiting immunomodulatory, anti-fibrotic and neutralizing activities over key local mediators, has promoted the IFP as an alternative source of MSC for cell-based therapy protocols. These complementary concepts have supported the growing notion of immune and inflammatory events participating in the pathogenesis of KOA, with the IFP/synovium complex engaging not only in amplifying local pathological responses, but also as a reservoir of potential therapeutic cell-based products. Consequently, the aim of this review is to outline the latest discoveries related with the IFP/synovium complex as both an active participant during KOA initiation and progression thus emerging as a potential target, and a source of therapeutic IFP-MSCs. Finally, we discuss how these notions may help the design of novel treatments for KOA through modulation of local cellular and molecular cascades that ultimately lead to joint destruction.

## Introduction

The infrapatellar fat pad (IFP), also known as Hoffa’s fat pad, is a cylinder-like piece of adipose tissue that sits posterior to the patella and fills the anterior knee compartment (Dragoo et al., 2012). Though the function of the IFP has not yet been fully defined, studies have shown that the IFP plays an important biomechanical role within the knee (Bohnsack et al., 2004; Gallagher et al., 2005). In addition, recent evidence has shown that the IFP in concert with the synovium participates in the pathogenesis and progression of various pathologies within the knee joint such as osteoarthritis (KOA) (Benito et al., 2005; Scanzello and Goldring, 2012; Sokolove and Lepus, 2013; Lieberthal et al., 2015; Felson et al., 2016; Favero et al., 2017; Mathiessen and Conaghan, 2017), given that these structures serve as sites of immune cell infiltration and origin of pro-inflammatory (*e.g.*, IFNγ, TNFα and IL1β) and articular cartilage degradative (*e.g.*, MMPs) molecules (Bondeson et al., 2010; Kalaitzoglou et al., 2017; Li et al., 2017). On the other hand, they may be related with repair attempts after injury, due to the presence of mesenchymal stem/stromal cells (MSCs) within both the IFP (IFP-MSC) (Garcia et al., 2016a; Tangchitphisut et al., 2016; Kouroupis et al., 2019a) and the synovium (sMSC) (Mizuno et al., 2018; To et al., 2019) exhibiting disease-modifying capacities (Caplan and Correa, 2011; Stagg and Galipeau, 2013; Uccelli and de Rosbo, 2015; Galipeau et al., 2016). Consequently, the IFP and synovium engage not only in amplifying local pathological responses, but also act as a reservoir of disease-modifying cellular products, promoting them as potential novel targets in joint disease (Attur et al., 2010).

IFP-MSCs have generated increased interest in recent literature due to their easy accessibility compared to other stem cell sources such as bone marrow and adipose tissue (AT), while displaying similar multipotency, growth potential, and immunomodulatory abilities (Sun et al., 2018). Their relative ease of isolation compared to bone marrow aspiration (thus removing the potential surgical complications seen with aspiration) have made them a popular resource for experimentation and regenerative medicine (Vilalta et al., 2008; Mizuno et al., 2012; Siciliano et al., 2016). However, because of its relatively newfound MSC population, current literature has re-focused on updating the knowledge of IFP anatomy, function, and most importantly its cellular composition beyond MSC. This has not only led to extensive investment in the IFP’s potential for regenerative medicine in Orthopedics, but also the role the IFP may play in certain pathological processes including KOA. For example, more established theories believe that the IFP communicates with the joint via the synovium and may play a role in cartilage and/or bone regeneration via the secretion of adipose tissue derived growth factors (Jiang et al., 2019). However, a shift in our understanding of the IFP anatomy and pathophysiology demonstrates not only that the IFP and the synovium constitute one structural and functional unit (Macchi et al., 2018), but that IFP-MSCs can regulate resident immune cell infiltration and resident macrophages thus acting as local immunomodulatory players.

Therefore, the goal of this review is to outline the latest developments of the IFP/synovium complex as a tissue that actively participates in joint homeostasis and disease, while harboring cellular elements that can be harnessed for therapeutic cell-based therapy protocols. In addition, updates regarding recent discoveries in anatomy, cellular composition, function, isolation and harvest, imaging, role in certain pathologies of the knee (most importantly modulation of inflammation in the joint), current therapeutic uses, and future perspectives and goals for IFP use will be discussed.

## Structure and Function of the IPF/Synovium Complex

### Anatomy

The IFP is located deep to the patella and occupies the space between the patellar tendon, femoral condyle, and tibial plateau. It attaches to the lower border of the patella, the intercondylar notch within the femur via the ligamentum mucosum, the periosteum of the tibia, and the anterior horn of both menisci (Gallagher et al., 2005). Of note, recent study of IFP anatomy has demonstrated previously undiscovered attachments to the deep quadriceps muscle, which may assist with IFP motion during walking (Woodley et al., 2012).

**FIGURE 1:**
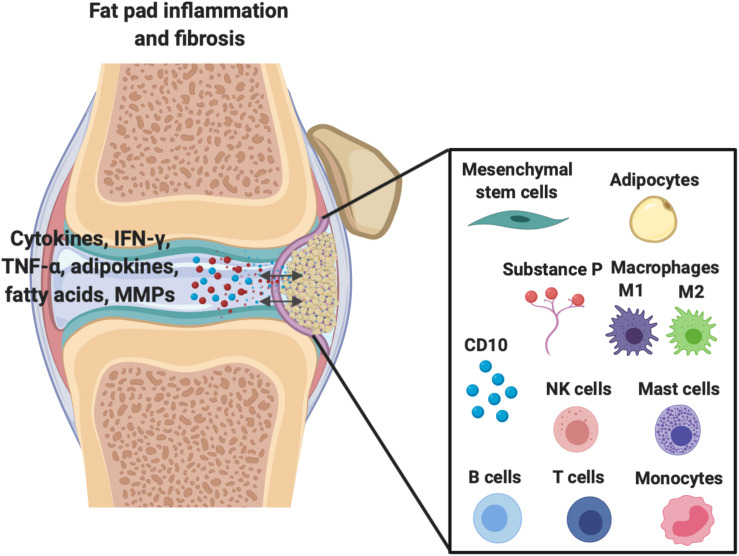
Demonstration of the extensive molecular cross-talk and important cellular components between the IFP and the synovium within the joint capsule which are responsible for inflammation/fibrosis.

Although the IFP is intracapsular, it remains extrasynovial despite its constant contact with the synovium (Clockaerts et al., 2010). Increasing evidence has demonstrated that the IFP develops as an outgrowth of the synovial tissue with regard to structure and functionality, which suggests extensive communication between the IFP and the synovium and joint capsule (Figure 1) (Ioan-Facsinay and Kloppenburg, 2013). Furthermore, Macchi et al. (2018) have concluded that the IFP and the synovium should be viewed as one anatomo-functional unit rather than two distinct structures that simply communicate with one another. The authors justified this definition because recent anatomical studies demonstrated the insertion of infrapatellar and medial synovial plicae directly onto the IFP, which suggests that the IFP may not be extrasynovial, but rather an extension of the synovium outside of the joint capsule (Macchi et al., 2018). Nevertheless, the intimate relationship between the synovium and IFP appears highly important in the release of growth factors and cytokines that help to regulate the molecular environment within the joint.

The extensive anastomotic vascular network near the IFP involves a combination of the superior and inferior geniculate arteries, the latter which passes through the IFP before supplying the patella. This matrix helps support and promote IFP-MSC proliferation, especially during injury and inflammation. It has also been hypothesized that this network is sufficient to protect the IFP during extensive surgical or arthroscopic procedures that lead to significant manipulation of the structure itself (Kohn et al., 1995).

Innervation to the IFP is just as extensive as its vasculature and typically traverses the same course across the entire tissue. Previous studies have confirmed that posterior articular branches from the tibial, saphenous, recurrent peroneal, and common peroneal nerve provide most of the innervation, however Gardner et al. recently described branches arising from the saphenous and obturator nerves as well (Freeman and Wyke, 1967; Kennedy et al., 1982). This collective peripheral sensory nociceptive innervation pattern (dense in parts of the IFP and synovium) is mediated by nerve fibers equipped with the neurotransmitter Substance P which runs separately but in parallel to sympathetic fibers and it is implicated in knee pain transmission. Additionally, within the IFP tyrosine hydroxylase (TH)-positive sympathetic fibers modulate nociception/pain signaling in sympathetic neurons, through interacting with Substance P-positive fibers (Dragoo et al., 2012; Brumovsky, 2016).

### Cellular Composition and Molecular Mediators

#### Infrapatellar Fat Pad

The most prevalent cell is the adipocyte, which is not only responsible for the IFP’s metabolism, but also endocrine and paracrine functions within the knee joint (Coelho et al., 2013; do Amaral et al., 2017). Importantly, adipose cells secrete cytokines, interferons, adipokines, and growth factors, all of which exerting local signaling effects on articular cartilage and synovial cells (Clockaerts et al., 2010).

As shown in Figure 1, other important cellular components of the IFP include fibroblasts, responsible for the production of extracellular matrix, and in less quantities resident monocytes, mast cells, lymphocytes, and perhaps most importantly macrophages (de Lange-Brokaar et al., 2012; Belluzzi et al., 2019; Kouroupis et al., 2019a). Barboza et al. (2017) have demonstrated that macrophages not only permanently reside within the IFP, but lie without phenotypic polarization as either classical M1 or alternative M2 variants until conditions promote their activation and subsequent conversion, such as inflammation.

**FIGURE 2:**
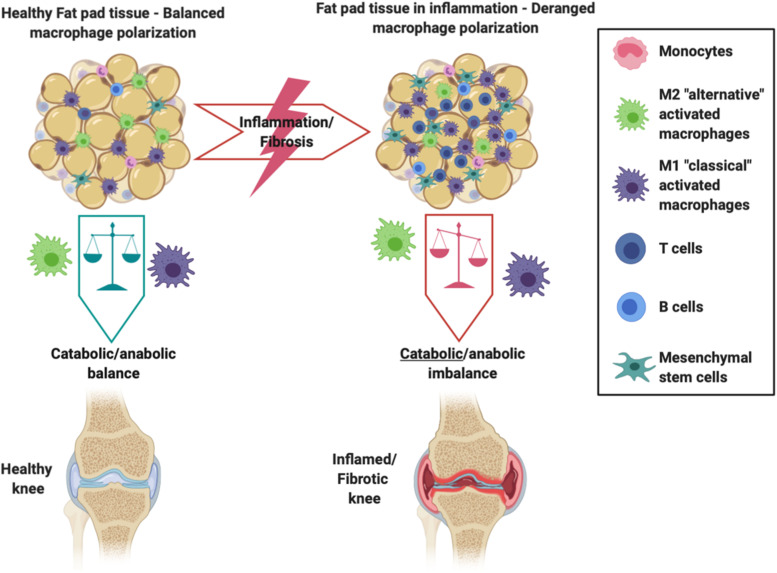
Schematic showing the intricate balance between M1 and M2 macrophages within the IFP and synovium. Differentiated M2 macrophages exhibit anti-inflammatory effects and preserve the health of the joint, whereas an imbalance favoring M1 macrophages promotes the IFP’s secretion of pro-inflammatory cytokines and catabolic factors that are seen within an inflamed/fibrotic knee.

Resident IFP macrophages are activated by a variety of interleukins and interferons secreted from other resident and infiltrating immune cells and adipose cells within the IFP. When converted to M1 macrophages, the IFP begins secreting vast amounts of pro-inflammatory cytokines, catabolic factors, and adipokines, and with prolonged periods of time, the IFP can also release pro-fibrotic mediators such as CTGF that may contribute to KOA progression (Figure 2) (Clockaerts et al., 2010). This occurs due to tight molecular crosstalk between synovial, IFP, and systemic inflammatory mediators. Consequently, these macrophages are now the target of studies assessing their release of pro-inflammatory molecular mediators (Kouroupis et al., 2019a). On the other hand, also shown in Figure 2, alternatively differentiated M2 macrophages exert anti-inflammatory effects, serving as counterbalance to their M1 cohorts by suppressing their proliferation and inflammatory signaling.

Finally, the IFP harbors a population of MSC (IFP-MSC), which will be discussed in detail later (see section “Synovium-Derived MSC”) as a potential therapeutic tool for cell-based therapy protocols.

#### Synovium

Within the intimal synovial lining reside predominantly two synovial cell types: type A (Macrophage-Like synoviocytes – MLS) and type B (Fibroblast-Like-Synoviocytes – FLS) (Tu et al., 2018). The type B synoviocytes, thought to be descendants of cells of mesodermal origin, are far more abundant and display typical fibroblast markers such as surface marker Thy-1 (CD90) and integrins like ICAM1 while secreting specialized matrix constituents including hyaluronan and Type IV and V collagens (Roelofs et al., 2017; Tu et al., 2018). Thus, it can be argued that type B synoviocytes more so than type A counterparts are responsible for maintenance of synovial homeostasis.

Type B synoviocytes are subject to cytokine and growth factor regulation, which can dictate a pro or anti-inflammatory state depending on which factors are expressed in the surrounding synovial environment (Orr et al., 2017). In a chronic inflammatory state, these cells primarily act in a pro-inflammatory role. For example, in patients with rheumatoid arthritis, fibroblasts have been shown to respond to and secrete a combination of TNF-alpha, IL-1, IL-6, and granulocyte-macrophage colony-stimulating factor (GM-CSF), while expressing a multitude of toll-like receptors in order to amplify T-Cell response to TLR activation (Ospelt, 2017). In regard to patients with OA, type B synoviocytes are particularly sensitive to TLR-2, TLR-3, and TLR-4 ligands due to the active expression of CD14, a co-receptor for TLRs (Nair et al., 2012). Type B synoviocytes also secrete a multitude of chemoattractants, including CCL2, CCL5, CCL8, CXCL5, and CXCL10, designed to attract monocytes and macrophages, both resident and peripheral in nature (Bartok and Firestein, 2010). Finally, upon stimulation of TLR-3, these synoviocytes produce large quantities of IL6, B-Cell Activating Factor and proliferation-inducing ligand (Mata-Essayag et al., 2001), promoting the maturation, survival, and antibody production by B cells (Bombardieri et al., 2011). These findings suggest that type B synoviocytes, though non-immune in nature, play a key role in autoimmune and OA disease development due to their inflammatory properties.

Type B synoviocytes can also produce a wide variety of anti-inflammatory factors such as TGF-beta, Type 1 interferons, VEG-F, indoleamine 2,3-dioxygenase (IDO enzyme), and certain prostaglandins, though some of these factors depending on concentration and exposure time may also be pro-inflammatory (Tu et al., 2018). However, the ability to harness the anti-inflammatory properties of these cells remains unknown. The recent proposition that type B synoviocytes may also contain multiple subtypes within the synovial lining which determine their secretory properties provides a future avenue for studies attempting to fully elucidate the role of these cells in arthritis development or modulation (Frank-Bertoncelj et al., 2017).

On the other hand, type A synoviocytes are far less known due to the limited number of these cells *in vivo* and their poor proliferative potential *in vitro*. They constitute resident macrophages, derived from both embryonic hematopoietic precursors and from bone marrow, although their definitive origin is still elusive (Tu et al., 2018). These resident macrophages need to be discriminated from monocytes/macrophages that extravasate into the synovium from peripheral circulation after injury or in disease. Nevertheless, it has been established that they have pro-inflammatory tendencies while exhibiting an intimate crosstalk with type B synoviocytes, especially in disease (Tu et al., 2018, 2019). Type A synoviocytes secrete soluble CD14, IL-1β, and TNFα, further potentiating the pro-inflammatory properties of type B synoviocytes and CD4 T helper cells. They also induce monocyte/macrophage-derived osteoclast activity via RANK-L secretion resulting in enhanced bone resorption (Yoshitomi, 2019). It is interesting to note given the above pro-inflammatory properties that the presence and activity levels of these tissue-resident macrophages significantly correlates with advanced stages of OA and poorer clinical outcome scores (Kriegova et al., 2018; Gomez-Aristizabal et al., 2019).

Similar to the IFP, the synovium contains a small population of cells compatible with MSC (sMSC), which will be discussed in detail in section “MSC-Induced Immunomodulation: Focus on Macrophage Polarization.”

#### IFP/Synovium Molecular Interactions

Beyond the proximity the IFP and synovium share, there are molecular interactions between both components that support their view as a single anatomical and functional unit. For instance, both IFP and adjacent synovium experience similar structural effects in KOA, including increased inflammatory infiltration, vascularization, and thickness (Favero et al., 2017). The IFP has been shown to release prostaglandin F2a (PGF2α), IL-6, IL-8, and TNFα, inducing a profibrotic effect on the synovial membrane (Bastiaansen-Jenniskens et al., 2013; Eymard et al., 2014). Specifically, Bastiaansen-Jenniskens et al. (2013) cultured human fibroblast -like synoviocytes (type B) obtained from OA patients in conditioned medium derived from IFP tissue with and without inhibitors of TGFβ/activin receptor-like kinase 5 or PGF2α for 4 days *in vitro*. IFP derived conditioned medium not only increased the migration and proliferation of synoviocytes but also resulted in profibrotic changes including Collagen production and *PLOD2* gene expression upregulation. Collagen production in synoviocytes was directly associated with secreted PGF2α levels in IFP derived conditioned medium. On the other hand, as the IFP is mainly composed of adipocytes, it results as a major source of various adipocyte-derived inflammatory mediators including lipids. Previous studies indicated that IFP-derived adipocytes, via secreted lipids, are able to modulate infiltrating macrophages and CD4^+^ T cells into the OA synovium (Ioan-Facsinay et al., 2013; Klein-Wieringa et al., 2013). In adipocyte-derived conditioned medium obtained from IFP, Ioan-Facsinay et al. (2013) identified free fatty acids that enhance CD4^+^ T cell proliferation and their capacity to produce IFN-γ. Additionally, free fatty acids secreted from IFP adipocytes can reduce the secretion of IL-12p40 cytokine by macrophages (Klein-Wieringa et al., 2013). According to previous studies (reviewed in Cooper and Khader, 2007), IL-12p40 is a chemoattractant molecule for macrophages, and which promotes inflammation and fibrosis. Furthermore, Mustonen et al. (2019) identified distinct fatty acid signature for IFP in OA and rheumatoid arthritis (RA) patients. Compared to RA, OA patients have higher total n-6, 20:4n-6 and 22:6n-3 polyunsaturated fatty acids (PUFA), and higher product/precursor ratios of n-3 PUFA. In general, n-6 PUFA such as 20:4n-6 (arachidonic acid) are precursors to pro-inflammatory mediators, whereas n-3 PUFA such as 22:6n-3 (docosahexaenoic acid) have anti-inflammatory/anti-catabolic effects (Brouwers et al., 2015). Overall, the major alterations in OA and RA joints compared to control healthy knees are an increase in monounsaturated fatty acids and a simultaneous decrease in n-6 PUFA, effects that should be further investigated in future studies (Mustonen et al., 2019).

Just as IFP influences synovium, Clements et al. (2009) demonstrated that extensive synovial proliferation and fibrosis led to marked loss of adipocytes within the IFP. Specifically, synovium secretion of pro-inflammatory cytokine IL-1β has been associated with catabolic effects in initiation and progression of OA. A previous study showed that exposure of IFP explants from OA patients to IL-1β *in vitro* result in secretion of large amounts of pro-inflammatory cytokines such as PTGS2, IL-1β, MCP-1, and IL-6. These effects can be partially ameliorated by a PPARα agonist (Clockaerts et al., 2012). Thus, recent literature has not only demonstrated extensive communication between both the IFP and synovium, but that this communication can accelerate development and progression of KOA, as elaborated below.

## IFP in the Pathogenesis of Knee OA – Potential New Target for Therapy

With the cellular composition of the IFP better elucidated and the occurrence of immune and inflammatory events within the IFP, its role in the pathophysiology of KOA is becoming the focus of multiple studies. For instance, Heilmeier et al. (2019) demonstrated that following ACL acute injury the IFP rapidly releases inflammatory cytokines that promote a sustained inflammatory response lasting for months. Consequently, various theories have emerged explaining the IFP’s role in the regulation of local inflammatory cascades including adipocytes, and more recently resident macrophages as key targets (in the development of post-traumatic OA). We next explore the strengths and limitations of each prevailing theory.

### IFP-Derived Adipocytes and Obesity Accelerate KOA Development

As previously discussed, adipocytes are capable of secreting certain molecular markers and products capable of initiating a local inflammatory response. Given that obesity represents a chronic inflammatory state, many studies have focused on the role of adipocytes as contributors for accelerated development of KOA (Balistreri et al., 2010; Bravo et al., 2019; Jiang et al., 2019). Consistent with this theory, the discovery of IL-1β and other pro-inflammatory cytokine production, together with matrix metalloprotease expression within KOA cartilage by adipocytes, suggests that the IFP may be intimately linked to KOA (Clockaerts et al., 2010; de Boer et al., 2012; Beekhuizen et al., 2013). Furthermore, leptin and adiponectin have been shown to be primarily secreted by IFP adipocytes into synovial fluid, with a key role influencing cartilage and synovial metabolism (Dumond et al., 2003; Toussirot et al., 2007). Therefore, the association of leptin to obesity and inflammation led to the belief that obesity itself plays a role in inducing IFP adipocyte inflammatory propagation and accelerated KOA progression (Ioan-Facsinay and Kloppenburg, 2013). Leptin has been shown to promote production of articular cartilage proteoglycans and collagen while stimulating insulin-like growth factor-1 and other growth factors that subsequently enhance chondrocyte proliferation (Bao et al., 2010, 2014). Lipid-mediated lipoxin A4, which can prevent cartilage degeneration in the knee, is also secreted by IFP adipocytes (Bastiaansen-Jenniskens et al., 2012; Gierman et al., 2013). Leptin facilitates the activation of immune cells, particularly M1 macrophages, via interferon release and nitric oxide production (Matarese et al., 2007). Moreover, recent literature suggests that obese patients with OA have either no difference in the number of M1 macrophages within the IFP, or may even have an increased number of M2 macrophages, compared to that of lean patients (de Jong et al., 2017). Lastly, even with M1 macrophages present within the IFP, the classic M1 macrophage mediated inflammation that usually occurs in abdominal adipose tissue as seen with obesity cannot be recapitulated, suggesting that IFP adipocytes are subject to distinct spatial-temporal metabolic regulation (Barboza et al., 2017).

An alternative mechanism by which obesity may affect the IFP during the progression of KOA is through altered joint mechanics. Ballegaard et al. (2014) have shown that obese patients with KOA demonstrated significantly increased inflammatory signaling within the IFP measured by contrast-enhanced perfusion variables on MRI. Cowan et al. (2015) also demonstrated that patients with patellofemoral OA have a greater IFP volume on MRI compared to healthy knees. Because the IFP resides in a tight anatomical space, the authors suggested that increased IFP volume was an inducer of inflammation, leading to secretion of synovial inflammatory factors (Clements et al., 2009). Therefore, in this alternate hypothesis, adipocyte induced inflammation within the joint may be due to factors other than obesity (King et al., 2013). However, OA also occurs in non-weight bearing joints such as the hand, suggesting that the metabolic effects of obesity may play a greater role than altered joint mechanics (Losina et al., 2011; Yusuf, 2012; Bliddal et al., 2014).

Overall, though there is an established link between obesity and KOA, the explanation that the IFP propagates KOA development because of its primarily adipocyte-based composition remains controversial. Because the IFP has a distinct environment compared to abdominal adipose tissue, the role of this specific adipocyte population in KOA remains unclear and warrants continued investigation, as obesity related features seen in visceral adipose tissue are not present within the IFP of KOA patients (de Jong et al., 2017).

### Role of IFP/Synovium Resident Macrophages

Adipocytes are not the only cellular component with potential to induce or enhance inflammation locally. The IFP and synovium are populated by macrophages, historically viewed as cells that maintain tissue homeostasis with crucial roles in early and late phases of response to injury, while more recently associated with various pathologies (Caspar-Bauguil et al., 2005; Mathis, 2013; Ginhoux and Jung, 2014). Macrophages have distinct origins resulting in significant heterogeneity, beyond the known M1 (classical pro-inflammatory) and M2 (alternative anti-inflammatory) polarization phenotypes (Ginhoux and Jung, 2014; Paul et al., 2015; Wu et al., 2020). A special population of tissue resident macrophages derive from embryonic precursors, exhibit self-renewal, and replenish after injury independently from circulating bone marrow-derived Ly6C^High^ monocytes (Ly6C^High^ is a murine marker with no current human ortholog identified) (Davies et al., 2011; Gentek et al., 2014; Ginhoux and Guilliams, 2016; Zhao et al., 2018). IFP and synovium show such resident populations with comparable immune cell profiles (Klein-Wieringa et al., 2016), also susceptible to polarize to M1 or M2 phenotypes depending on the status of the joint (Barboza et al., 2017; Sun et al., 2017; Tu et al., 2018; Wu et al., 2020).

Resident M1 pro-inflammatory macrophages are theorized to be an important driver of the host low grade chronic inflammatory state (Mathis, 2013; Kandahari et al., 2015). In fact, patients with KOA show a propensity for the M1 classical phenotype within the IFP/synovium complex, resulting in cytokine, interferon, and TNF-alpha secretion (Klein-Wieringa et al., 2011; Wu et al., 2020). Recent evidence suggests that activation of the mammalian target of rapamycin (Kuptniratsaikul et al., 2009) pathway also plays a role in M1 macrophage polarization and progression of KOA in animal models (Fernandes et al., 2020). Nevertheless, the existence of resident macrophages within the IFP exhibiting an M1 phenotype independent of the presence of local inflammation confirms their potential participation as initiators of KOA (Wu et al., 2020). Thus, the propagation of KOA is not reliant solely on immune cell extravasation, but rather on resident cells from within the IFP/synovium complex, though the precise turning point that leads to KOA still remains unknown.

### Immune Infiltration to the IFP/Synovium

The aforementioned molecular markers that induce pro-inflammatory states do so in part by promoting extravasation of circulating immune cells into the IFP and synovium. The secretion of related prostaglandins, as well as IL-6 and IL-8 promote the extravasation of immune cells by attracting lymphocytes to the endothelium promoting their migration into the surrounding IFP and synovium (Schnoor et al., 2016). Substance P, a product of nociceptive nerve fibers that transmits pain signals while also modulates local inflammatory processes (*i.e.*, neurogenic inflammation), has also been shown to induce vasodilation of peripheral vessels, thus promoting the extravasation of immune cells from peripheral circulation into surrounding tissue (Clockaerts et al., 2010).

Apinun et al. (2016) described the presence of peripheral CD8 T cells, macrophages, B cells, and mast cells within the IFP of patients with OA undergoing TKA. According to the authors, the infiltration of these cells trended with disease severity (patients with severe radiographic KOA had more CD8 T cell infiltration than patients with mild KOA), thereby leading the authors to conclude that the infiltration of circulating immune cells to the IFP and synovium contribute to disease progression and severity. In addition, Klein-Wieringa et al. (2016) showed that peripheral CD4 T cells also infiltrate the IFP and synovium in a severely osteoarthritic population, and their presence correlated with pain scores (*R* = 0.53, *p* < 0.01, *N* = 76 patients). Thus, pro-inflammatory cells within the IFP and synovium not only promote localized inflammation with resident immune cells, but also promote extravasation of circulating ones potentiating the inflammatory process that are associated with poorer clinical and radiographic outcomes.

### Clinical Correlation: Imaging to Assess IFP Changes During OA Progression

The IFP is best visualized on non-contrast magnetic resonance imaging (MRI) in the sagittal plane and intensity of signal alterations have recently been correlated with anterior knee pain and cartilage loss by Han et al. (OR 1.23, *p* < 0.05, *N* = 374), supporting the link between changes in IFP and KOA development (Hill et al., 2007; Roemer et al., 2009; Han et al., 2016). Though non-contrast enhanced MRI is the gold standard, contrast-enhanced MRI imaging has recently been employed to show correlations between histological synovial infiltrate and hyperplasia and KOA progression (*R* = 0.63, *p* < 0.001, *N* = 30) (Loeuille et al., 2011). Crema et al. (2013) have shown that peri-patellar synovial thickness on non-contrast-enhanced MRI images could be the culprit for KOA related pain and not the changes in signal alterations within the IFP itself.

Interestingly, the size of the IFP may play an important role in KOA risk and symptom development and intensity. Pan et al. (2015) demonstrated that decreased IFP volume in older women compared to men was significantly associated with increased total knee pain, pain at rest and during movement, and cartilage damage. However, the authors also found that total IFP maximal area appears to have a protective role for knee symptoms in older adult females, but not men. IFP signal intensity was later linked to size of IFP by Han et al. (2016), which provides support for continued use of non-contrast enhanced MRI as the gold standard. Recently, Fontanella et al. (2019) reported changes in the morphometry (i.e., reduced volume, depth, and femoral and tibial arc lengths) and increase of the MRI hypointense signal in the IFP from patients with moderate and end-stage KOA compared to healthy controls. Despite contrasting results from various groups, the description of morphological changes in the IFP by MRI warrants continued investigation into how imaging may play a future in predicting KOA risk or progression.

## IFP/Synovium as a Source of MSC for Cell Therapy

### IFP-MSC

In 1996 a pioneering study by Maekawa et al. (1996) firstly described a type of fibroblastic cells possessing ‘stem cell-like’ characteristics in synovial tissue near the IFP. Those cells reside mostly in the perivascular space surrounding vessels of small caliber and involved in the fibronectin and laminin production. Recent studies have isolated and phenotypically characterized IFP-MSC positive for CD9, CD10, CD13, CD29, CD44, CD49e, CD59, CD73, CD90, CD105, CD106, CD146, CD166, NG2, and CXCR4 markers, while negative for CD34, CD56, CD200, CD271, 3G5, LepR and STRO-1 markers (Wickham et al., 2003; Khan et al., 2008; Garcia et al., 2016a; Hindle et al., 2017; Kouroupis et al., 2019a). IFP-MSC characteristically have low or no HLA-DR expression, yet a total absent expression of co-stimulatory molecules CD40, CD80, and CD86 (Garcia et al., 2016a; Kouroupis et al., 2020). In a recent study, Hindle et al. identified two distinct IFP-MSC subpopulations within the IFP, characterized as pericytes (CD31^–^CD45^–^CD34^–^CD146^+^) and adventitial cells (CD31^–^CD45^–^CD34^+^CD146^–^), representing 3.8 and 21.2% of the IFP stromal vascular fraction, respectively (Hindle et al., 2017).

In general, IFP-MSC have comparable proliferative potential to other MSC types (Dragoo et al., 2003; Jurgens et al., 2009). In comparative studies, IFP-MSC were reported to possess similar growth kinetics to bone marrow-derived MSC (BM-MSC) (English et al., 2007) and higher proliferation to donor-matched synovial fluid-MSC (Garcia et al., 2016a). However, in order to generate clinically relevant cell numbers, IFP-MSC growth rate can be accelerated by various *in vitro* culturing conditions such as human platelet lysate (hPL) or chemically-reinforced (Ch-R) media expansion, serum and growth factor (TGF-β and FGF-2) stimulation and hypoxia exposure (Marsano et al., 2007; Khan et al., 2008; Buckley and Kelly, 2012; Liu et al., 2012; O’HEireamhoin et al., 2013). Importantly, our group recently showed that hPL and Ch-R formulations can effectively replace FBS to expand IFP-MSC, enhancing phenotypic and functional attributes (Kouroupis et al., 2020).

IFP-MSC multipotentiality toward chondrogenic, osteogenic, and adipogenic lineages has been demonstrated by previous studies (reviewed in Sun et al., 2018). However, there is evidence showing that MSC differentiation capacity is strongly related to the tissue of origin. Therefore, due to the intra-articular localization of IFP tissue, and their anatomical proximity to articular cartilage, it is not surprising that IFP-MSCs exhibit strong chondrogenic differentiation capacity both *in vitro* and *in vivo* (Dragoo et al., 2003; Khan et al., 2008; Lee et al., 2008; Jurgens et al., 2009; Buckley et al., 2010; Almeida et al., 2014, 2015, 2016; Liu et al., 2014; Ye et al., 2014). Specifically, *in vitro* IFP-MSC show stronger chondrogenic differentiation capacity than adipose- derived-, BM-, and UC-MSC (Ding et al., 2015). Others however report that they possess at least comparable chondrogenic capacity to BM-MSC (English et al., 2007) but inferior to native chondrocytes and perivascular IFP-MSC (Marsano et al., 2007; Vinardell et al., 2011; Garcia et al., 2016b; Hindle et al., 2017). On this basis, studies have shown that heterogenous IFP-MSC selection for specific subpopulations may result in further enhanced chondrogenic differentiation capacity. Moreover, perivascular IFP-MSC (CD31^–^CD45^–^CD34^–^CD146^+^) generate significantly more extracellular matrix than heterogenous “crude” IFP-MSC cultures (Hindle et al., 2017). Also, others reported the positive correlation of CD49c expression of donor-matched chondrocytes, BM-MSC, FP-MSC, and synovial fluid MSC with their chondrogenic capacities *in vitro* (*R* = 0.2, *p* < 0.018, *N* = 5 samples) (Garcia et al., 2016b). In *in vivo* settings, freshly isolated uncultured CD44^+^ IFP-MSC seeded into a TGF-β3 ECM-derived scaffold and subcutaneously implanted in nude mice, are capable of producing a cartilage-like tissue rich of sGAG and Collagen type II (Almeida et al., 2015). Therefore, the selection of specific IFP-MSC subpopulations may result in improved *in vivo* chondrogenesis.

Given their high proliferation rate and superior chondrogenic differentiation capacity, IFP-MSC may be considered a suitable candidate cell to engineer cartilaginous constructs to resurface focal defects or even an entire OA joint (Liu et al., 2014; Ye et al., 2014; Prabhakar et al., 2016). In that regard, Liu et al. (2014) showed that IFP-MSC obtained from both healthy and OA individuals and cultured on PLLA fiber membranes for 6 weeks can generate robust, flexible cartilage-like grafts of clinically relevant dimensions (≥2 cm in diameter). Of note, the authors did note that donor age variability may affect the robustness of the cultured IFP-MSCs, supporting the idea that the outcome of future IFP-MSC treatments may be substantially different in certain patient populations.

However, the main limitation of MSC-based cartilage constructs is that they progress in differentiation reaching an ultimate hypertrophic phenotype and finally undergoing endochondral ossification *in vivo* (Farrell et al., 2009, 2011; Scotti et al., 2013; Correa et al., 2015; Feng et al., 2018). To overcome this limitation, co-culture of IFP-MSC with articular chondrocytes in hybrid structures result in a phenotypically stable layer of articular cartilage with reduced mineralization upon implantation in nude mice for 8 weeks (Mesallati et al., 2015). The same group demonstrated that self-assembled IFP-MSC on top of articular cartilage agarose gels result in higher accumulation of sGAG and therefore strongly enhance the development of articular cartilage constructs (Mesallati et al., 2017). Although, articular cartilage tissue engineering is a promising approach, a significant barrier is the generation of constructs with clinically relevant dimensions and in a time/cost efficient manner ready-to-use for *in vivo* implantation, especially for large compromised surfaces such as in OA. In more simplified approaches, IFP-MSC are directly injected intra-articularly solely or embedded in hydrogel-based delivery systems with and without growth factors. Toghraie et al. (2011) showed that a single dose of intra-articularly injected IFP-MSC result in decreased cartilage degeneration, osteophyte formation, and subchondral sclerosis 20 weeks later in a rabbit OA model. Recently, Muttigi et al. (2018) directly injected IFP-MSC embedded in matrillin-3 (an essential ECM component of cartilage) and 2% hyaluronic acid in an osteochondral defect rat model, with the reasoning that Matrilin-3 alone enhances Collagen II and aggrecan expression in chondrocytes while downregulating matrix degrading enzymes such as matrix metalloproteinase-13 (MMP-13). According to the authors, Matrillin-3, when is co-delivered with IFP-MSC, indeed resulted in greatly enhanced Collagen type II and aggrecan productions, whereas the regenerated defect site possesses similarities with native cartilage (thickness, chondrocyte clustering, and hyaline-like morphology). Overall, IFP-MSC due to their advantageous intra-articular anatomical localization and ease for harvesting along with their enhanced chondrogenic capacity may be an attractive approach for addressing articular cartilage degeneration in OA.

Our group recently reported that intra-articularly injected CD10-rich IFP-MSC reverted induced synovitis and IFP fibrosis in rats which are seen in early OA (Kouroupis et al., 2020). Interestingly, the degree of *in vivo* efficacy is associated with the degree of expression of CD10 and degradation of Substance P, a local mediator of transmitting pain signals and regulator of neurogenic inflammation.

### Synovium-Derived MSC

Located within the synovial intima and sub-intima lie distinct MSC populations similar to IFP-MSC, with an origin still debated (Li et al., 2019; Sivasubramaniyan et al., 2019). Though not populous within the tissue, these cells maintain high proliferative capacity and can differentiate into osteoblasts, adipocytes, and chondrocytes *in vitro* (Ferro et al., 2019). The origin of synovium-derived MSC (sMSC) in the synovial lining is still not fully defined, with groups supporting the notion of MSC infiltrating from resident vasculature or even from the neighboring bone marrow. However, two recently reports strongly support the hypothesis of the embryonic origin of synovium at the joint interzone, by showing that single or double positive Prg4-lineage and Gdf5-lineage cells, and associated sMSC as contributors to tissue homeostasis and repair in adult life (Decker et al., 2017; Roelofs et al., 2017).

Besides a similar overall immunophenotype of sMSC compared with other MSC (De Bari et al., 2001; Sakaguchi et al., 2005; Hermida-Gomez et al., 2011), CD271, a highly expressed markers in freshly isolated BM-SMC, is absent in healthy sMSC (Karystinou et al., 2009), yet expressed in cells isolated from OA patients (Hermida-Gomez et al., 2011). A topographic analysis of synovium and a full phenotypic description of sMSC are presented in our previous review (Kouroupis et al., 2019b). According to reports, sMSC show a greater proliferation rate and stronger chondrogenic capacity than BM- and adipose-derived MSC whereas they exhibit a reduced hypertrophic differentiation potential (Kubosch et al., 2018).

Previous studies have demonstrated that sMSC and IFP-MSC show comparable chondrogenic differentiation capacity (Mochizuki et al., 2006). In a comparative study of three different MSC types, Mochizuki et al. indicated that sMSC and IFP-MSC have similar chondrogenic capacity between older and younger donors but higher compared to donor-matched subcutaneous fat-derived MSC (Mochizuki et al., 2006). However, Vinardell et al. (2012) reported that when sMSC and IFP-MSC are embedded in agarose hydrogel constructs and chondrogenic induced for 49 days *in vitro*, sMSC accumulate higher levels of sGAG and Collagen than IFP-MSC. Similarly, to IFP-MSC, sMSC expansion in hPL medium result in increased proliferation rate but lower chondrogenic capacity compared to sMSC grown in the presence of FBS (Nimura et al., 2008).

In preclinical settings, Koizumi et al. (2016) isolated sMSC from both OA and RA patients and assessed their cartilage repair capacity using a scaffold-free tissue engineering approach. Interestingly, 8 weeks post-implantation both OA or RA sMSC-treated groups showed hyaline cartilage-like repair and in general higher histological scores compared to the untreated rats. In addition, various studies using animal sMSC (rabbit-, murine-, equine-, porcine-harvested synovium) showed that sMSC groups are superior to control groups in treating full thickness chondral lesions (To et al., 2019). Therefore, sMSC show good reparative capacity of chondral lesions and no adverse effects after implantation *in vivo*.

### MSC-Induced Immunomodulation: Focus on Macrophage Polarization

**FIGURE 3:**
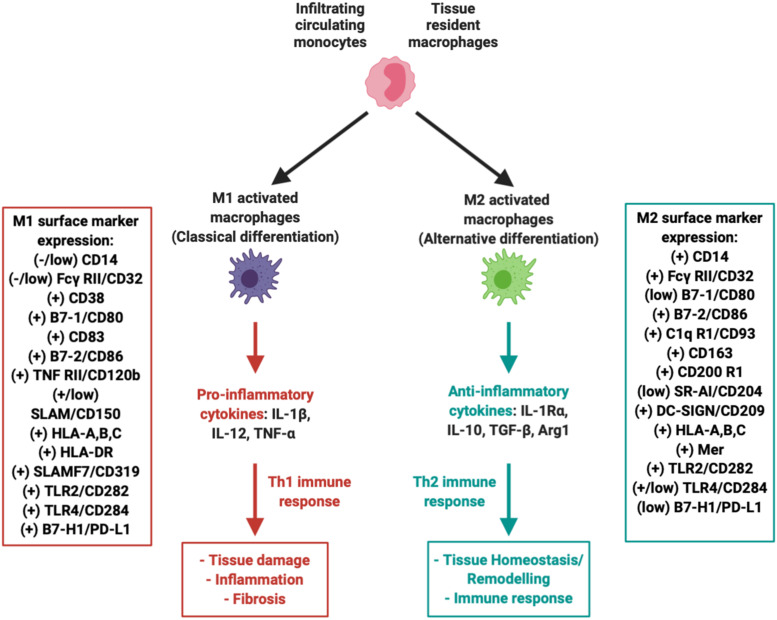
Macrophages within the IFP can polarize into M1 (pro-inflammatory) or M2 (anti-inflammatory, tissue repair) variants. Both variants are associated with a multitude of unique surface markers with varying levels of expression that allow for proper identification.

During early phases of OA, both IFP and synovium become infiltrated by immune cells, including T cells, B cells, monocytes/macrophages, and mast cells (Pelletier et al., 2001; Sokolove and Lepus, 2013; Ioan-Facsinay and Kloppenburg, 2017; Kalaitzoglou et al., 2017). These infiltrates complement the local resident cells, especially macrophages, which as seen in Figure 3 polarize into a pro-inflammatory classical M1 phenotype (discussed above in section “Role of IFP/Synovium Resident Macrophages”). MSC exerts immunomodulatory effects, simultaneously influencing multiple immune cells through different mechanisms including cell-cell contact, soluble factors, and released extracellular vesicles (*e.g.*, exosomes). The specific effects of MSC on T cells, B cells and other immune cells have been reviewed extensively elsewhere (Djouad et al., 2005; Hagmann et al., 2013). Herein, we emphasize on the current knowledge regarding the interactions with macrophages given their pivotal role in the initiation and progression of the disease as source of inflammatory and degradative mediators.

Numerous studies have explored macrophages as potential therapeutic targets, including their pharmacological depletion from synovium and IFP and manipulation of their phenotype [reviewed in Fernandes et al. (2020) and Wu et al. (2020)]. Initial evidence suggests that polarization of macrophages back to an alternative anti-inflammatory M2 phenotype can be induced. M2 macrophages represent the other extreme in terms of functionality, as they play a major role in local tissue repair by secreting low levels of anti-inflammatory cytokines such as IL-10 at a much more accelerated rate compared to unpolarized “naïve” resident macrophages (Figure 3) (Zeyda et al., 2007; Fernandes et al., 2020). In fact, it has been proposed that MSC can indeed promote M2 macrophage polarization *in vitro* (Harrell et al., 2019). Furthermore, our group recently reported the switch of IFP macrophages from an M1 to an M2 phenotype *in vivo*, after a single intra-articular injection of a subset of BM-MSC (CD146^+^) in rats with induced synovitis and IFP fibrosis (Bowles et al., 2020).

The effects of IFP-MSC in macrophage polarization are far less defined. Nevertheless, it has been described that Substance P within IFP actively participates in immune responses and inflammatory cascades (*i.e.*, neurogenic inflammation), enhancing the migration of monocytes to sites of inflammation (Mashaghi et al., 2016; Spitsin et al., 2017; Suvas, 2017). Relatedly, our group recently reported that upon exposure to a pro-inflammatory environment (Kouroupis et al., 2019a) and when manufactured under regulatory-compliant conditions (Kouroupis et al., 2020), IFP-MSC become enriched for CD10/neprilysin, an ectopeptidase that efficiently degrades Substance P both *in vitro* and *in vivo*. The resulting CD10-rich IFP-MSC exhibit an innate ability to selectively migrate to areas of active synovitis, reverse inflammation and fibrosis of synovium and IFP. Interestingly, these effects are directly related with the level of positivity for CD10 (Kouroupis et al., 2020). Furthermore, Substance P has been reported to induce the differentiation of pro-inflammatory macrophages into a special phagocytic M2 phenotype (M2^SP^), different from previously reported M2a and M2c subphenotypes (Lim et al., 2017).

### Efforts to Translate Pre-clinical Findings Into Clinical Protocols

MSC-based therapy to treat OA has received attention based on promising pre-clinical reports. Various cell sources have been successfully used in early-phase clinical trials, including bone marrow (Orozco et al., 2014; Vega et al., 2015; Soler et al., 2016), umbilical cord (Matas et al., 2019), and adipose-derived stromal vascular fraction (Garza et al., 2020). A recent systematic review summarizing available studies testing intra-articular MSC therapy for OA and chondral defects concluded that the therapy is safe with clinical and in some cases imaging improvement (McIntyre et al., 2018). Synovial and IFP-derived MSC are starting to be explored clinically, yet only initial data is available.

In a pioneering case control study, a mean of 1.89 million (range, 1.2–2.3 × 10^6^) IFP-MSC with platelet-rich plasma (PRP) were intra-articularly injected in OA patients after arthroscopic debridement, and controlled against debridement + PRP alone (Koh and Choi, 2012). Patients were followed for up to 12–18 months, reporting no adverse effects and a significant improvement in Patient Reported Outcome Measurements (PROMs) including Lysholm score, visual analog scale (VAS), and Tegner activity were noted in the study group compared with control cohorts. However, Koh et al. (2013) did note some limitations, namely that the control group was significantly different in terms of baseline radiographic and chondral lesion severity, as well as the small sample size with a focus on severe KOA patients.

The same team then in a 24–26-month follow-up study demonstrated that the significant decrease of the Western Ontario and McMaster Universities Osteoarthritis Index (WOMAC) is directly related to the amount of injected IFP-MSC (Koh et al., 2013). The authors also found that study patients demonstrated significantly improved cartilage whole-organ MRI scores that correlated strongly with decreased pain and improved function (*R* = −0.588 and −0.0536 respectively, *p* < 0.05, *N* = 18). Collectively, these initial results indicate the positive effect of intra-articularly injected IFP-MSC in reducing pain and improving knee function in OA patients, when compared with arthroscopic debridement and PRP alone. However, in addition to the various limitations acknowledged by the authors, the study population only included older patients with severe KOA, thus warranting further clinical investigation to determine the efficacy of the procedure as well as it’s applicability to a broader clinical population.

Similarly, sMSC yielded encouraging results in treating symptomatic chondral lesions in patients. Sekiya et al. (2015) expanded sMSC with autologous human serum and intra-articularly injected them to treat femoral condyle chondral lesions in 10 patients. For an average follow-up of 52 months, histologic analyses indicated hyaline and fibrous cartilage formation paralleled by improved Lysholm scores (Sekiya et al., 2015). In another study, autologous sMSC scaffold-free constructs were implanted in five patients to treat 1.5–3.0 cm^2^ chondral lesions (Shimomura et al., 2018). Forty-eight weeks post-implantation all patients achieved defect filling with tissue integration whereas histological analysis indicated strong cartilaginous tissue formation in all patients, with few spindle-shaped fibroblast-like cells localized only at the new-formed cartilage superficial zone. No adverse effects and significantly clinical improvements were reported at a 24-month follow-up.

These preliminary clinical studies indicated for both IFP-MSC and sMSC the overall significant improvement in cartilage repair without any complications for the patients treated. Nevertheless, the small number of patients involved and the potential confounding effect of parallel products (e.g., PRP) requires the design of prospective randomized, controlled trials to establish efficacy beyond the established safety.

## Future Perspectives

The involvement of immune and inflammatory events within the synovium and IFP during early KOA has led to changes in our thinking of the disease and potential treatment approaches. Furthermore, the identification of resident and infiltrating macrophages as key modulators of those events presents a novel therapeutic target in the treatment of KOA. The ability of locally delivered IFP-MSCs to regulate synovial/IFP inflammation and fibrosis then becomes a promising therapeutic alternative to mitigate disease progression of the disease. Nevertheless, more information is required to solidly connect MSC local effects, macrophage phenotypic polarization and inflammation/fibrosis control with a durable effect limiting KOA progression.

Moreover, one critical aspect to understanding the impact of macrophages in the IFP is to dissect the molecular, cellular, and genetic identities of the heterogeneous tissue. Critically, over the past decade, numerous technologies allowing single-cell RNA sequencing (scRNA-seq) have emerged to provide unprecedented ability to examine gene expression profiles at the single cell level (Svensson et al., 2018). In general, these techniques allow the deconvolution of a heterogeneous tissue into specific cell types and an examination of their abundance. Furthermore, subtle differences between cells of similar lineages can be distinguished on the basis of just a few gene expression changes. Finally, there is the ability to compare cellular profiles and gene expression across samples, conditions, or groups of individuals. While efforts are currently underway to dissect the cellular complexity of tissues throughout the body including articular cartilage during OA progression (Regev et al., 2017; Ji et al., 2019), the IFP is noticeably absent from these efforts. To our knowledge, there is no existing high throughput single cell expression profile of the IFP, either in its nascent state or following injury or in chronic disease. Given its role in the immune responses to these conditions, this remains a topic of importance moving forward in characterizing its importance.

Mechanistically, Substance P targeting and degradation by CD10-rich MSC could become a mechanism to disrupt the sustained chronic inflammation within the IFP and the transmission of nociception signals from the knee to the central nervous system. As such, the reduction of Substance P^+^ nerve fibers within IFP may possibly be related to control of KOA’s most prevalent clinical presentation, joint pain.

Finally, an emerging approach results from the description of extracellular vesicles (*e.g.*, exosomes) released by MSC, and their involvement in the therapeutic activities of the cells. For instance, our previous reports indicate comparable effects between cells and their supernatant in terms of their ability to degrade Substance P. These observations may support the idea of a “cell-free” product that may recapitulate the therapeutic effects of their parental cells, with manufacturing advantages as previously described (Pachler et al., 2017; Rohde et al., 2019; Witwer et al., 2019). In fact, the potential use of exosome-based cell-free products has already sparked multiple pre-clinical studies assessing potential clinical translation of cell-free products with encouraging results (Kordelas et al., 2014; Mendicino et al., 2014; Karnieli et al., 2017; Hu et al., 2019; Cai et al., 2020; Jiang et al., 2020; Meng and Qiu, 2020).

## Conclusion

The knowledge accrued over the last decade regarding the IFP has led to important discoveries that elucidate its role beyond that of a vascular tissue with a biomechanical role in the anterior compartment of the knee. The proposition of the IFP and the synovium functioning as a single unit and the now recognized tight molecular crosstalk between both structures has been shown to promote resident immune cells, immune cell infiltration, and the subsequent production of articular cartilage degradative molecules associated with the propagation of various knee pathologies such as KOA. On the other hand, the presence of IFP-MSC and sMSC suggest that the IFP and synovium act as a reservoir of therapeutic cellular products engaged with repair after exposure to inflammation and subsequent injury. These MSC have also been shown to modulate macrophage phenotypic polarization in favoring immunomodulatory conditions.

The ability of local MSC to regulate synovial/IFP inflammation and fibrosis poses a promising therapeutic target to mitigate disease progression. Therefore, the IFP presents an important target for limiting joint disease progression. More information is required to better understand the connection between the local MSC population and macrophage phenotypic polarization as it relates to controlling the propagation of inflammation/fibrosis and subsequent progression of KOA.

## Author Contributions

All authors listed have made a substantial, direct and intellectual contribution to the work, and approved it for publication.

## Conflict of Interest

DC is a paid consultant of Lipogems USA, LLC. The remaining authors declare that the research was conducted in the absence of any commercial or financial relationships that could be construed as a potential conflict of interest.

## References

[B1] Almeida H. V. Cunniffe G. M. Vinardell T. Buckley C. T. O’Brien F. J. Kelly D. J. (2015). Coupling freshly isolated CD44(+) infrapatellar fat pad-derived stromal cells with a TGF-beta3 eluting cartilage ECM-derived scaffold as a single-stage strategy for promoting chondrogenesis. *Adv. Healthc. Mater.* 4 1043–1053. 10.1002/adhm.201400687 25656563

[B2] Almeida H. V. Eswaramoorthy R. Cunniffe G. M. Buckley C. T. O’Brien F. J. Kelly D. (2016). Fibrin hydrogels functionalized with cartilage extracellular matrix and incorporating freshly isolated stromal cells as an injectable for cartilage regeneration. *Acta Biomater.* 36 55–62. 10.1016/j.actbio.2016.03.008 26961807

[B3] Almeida H. V. Liu Y. Cunniffe G. M. Mulhall K. J. Matsiko A. Buckley C. T. (2014). Controlled release of transforming growth factor-beta3 from cartilage-extra-cellular-matrix-derived scaffolds to promote chondrogenesis of human-joint-tissue-derived stem cells. *Acta Biomater.* 10 4400–4409. 10.1016/j.actbio.2014.05.030 24907658

[B4] Apinun J. Sengprasert P. Yuktanandana P. Ngarmukos S. Tanavalee A. Reantragoon R. (2016). Immune mediators in osteoarthritis: infrapatellar fat pad-infiltrating CD8+ T Cells are increased in osteoarthritic patients with higher clinical radiographic grading. *Int. J. Rheumatol.* 2016:9525724. 10.1155/2016/9525724 PMC5192329 28070192

[B5] Attur M. Samuels J. Krasnokutsky S. Abramson S. B. (2010). Targeting the synovial tissue for treating osteoarthritis (OA): where is the evidence? *Best Pract. Res. Clin. Rheumatol.* 24 71–79. 10.1016/j.berh.2009.08.011 20129201

[B6] Balistreri C. R. Caruso C. Candore G. (2010). The role of adipose tissue and adipokines in obesity-related inflammatory diseases. *Mediat. Inflamm.* 2010:802078. 10.1155/2010/802078 PMC2910551 20671929

[B7] Ballegaard C. Riis R. G. Bliddal H. Christensen R. Henriksen M. Bartels E. M. (2014). Knee pain and inflammation in the infrapatellar fat pad estimated by conventional and dynamic contrast-enhanced magnetic resonance imaging in obese patients with osteoarthritis: a cross-sectional study. *Osteoarthr. Cartilage* 22 933–940. 10.1016/j.joca.2014.04.018 24821663

[B8] Bao J. P. Chen W. P. Feng J. Hu P. F. Shi Z. L. Wu L. D. (2010). Leptin plays a catabolic role on articular cartilage. *Mol. Biol. Rep.* 37 3265–3272. 10.1007/s11033-009-9911-x 19876764

[B9] Bao J. P. Jiang L. F. Chen W. P. Hu P. F. Wu L. D. (2014). Expression of vaspin in the joint and the levels in the serum and synovial fluid of patients with osteoarthritis. *Int. J. Clin. Exp. Med.* 7 3447–3453. 25419381 PMC4238502

[B10] Barboza E. Hudson J. Chang W. P. Kovats S. Towner R. A. Silasi-Mansat R. (2017). Profibrotic infrapatellar fat pad remodeling without m1 macrophage polarization precedes knee osteoarthritis in mice with diet-induced obesity. *Arthritis Rheumatol.* 69 1221–1232. 10.1002/art.40056 28141918 PMC5449220

[B11] Bartok B. Firestein G. S. (2010). Fibroblast-like synoviocytes: key effector cells in rheumatoid arthritis. *Immunol. Rev.* 233 233–255. 10.1111/j.0105-2896.2009.00859.x 20193003 PMC2913689

[B12] Bastiaansen-Jenniskens Y. M. Clockaerts S. Feijt C. Zuurmond A. M. Stojanovic-Susulic V. Bridts C. (2012). Infrapatellar fat pad of patients with end-stage osteoarthritis inhibits catabolic mediators in cartilage. *Ann. Rheum. Dis.* 71 288–294. 10.1136/ard.2011.153858 21998115

[B13] Bastiaansen-Jenniskens Y. M. Wei W. Feijt C. Waarsing J. H. Verhaar J. A. Zuurmond A. M. (2013). Stimulation of fibrotic processes by the infrapatellar fat pad in cultured synoviocytes from patients with osteoarthritis: a possible role for prostaglandin f2alpha. *Arthritis Rheum.* 65 2070–2080. 10.1002/art.37996 23666869

[B14] Beekhuizen M. Gierman L. M. van Spil W. E. Van Osch G. J. Huizinga T. W. Saris D. B. (2013). An explorative study comparing levels of soluble mediators in control and osteoarthritic synovial fluid. *Osteoarthr. Cartilage* 21 918–922. 10.1016/j.joca.2013.04.002 23598178

[B15] Belluzzi E. Stocco E. Pozzuoli A. Granzotto M. Porzionato A. Vettor R. (2019). Contribution of infrapatellar fat pad and synovial membrane to knee osteoarthritis pain. *Biomed. Res. Int.* 2019:6390182. 10.1155/2019/6390182 PMC6462341 31049352

[B16] Benito M. J. Veale D. J. FitzGerald O. van den Berg W. B. Bresnihan B. (2005). Synovial tissue inflammation in early and late osteoarthritis. *Ann. Rheum. Dis.* 64 1263–1267. 10.1136/ard.2004.025270 15731292 PMC1755629

[B17] Bliddal H. Leeds A. R. Christensen R. (2014). Osteoarthritis, obesity and weight loss: evidence, hypotheses and horizons - a scoping review. *Obes. Rev.* 15 578–586. 10.1111/obr.12173 24751192 PMC4238740

[B18] Bohnsack M. Wilharm A. Hurschler C. Ruhmann O. Stukenborg-Colsman C. Wirth C. J. (2004). Biomechanical and kinematic influences of a total infrapatellar fat pad resection on the knee. *Am. J. Sports Med.* 32 1873–1880. 10.1177/0363546504263946 15572315

[B19] Bombardieri M. Kam N. W. Brentano F. Choi K. Filer A. Kyburz D. (2011). A BAFF/APRIL-dependent TLR3-stimulated pathway enhances the capacity of rheumatoid synovial fibroblasts to induce AID expression and Ig class-switching in B cells. *Ann. Rheum. Dis.* 70 1857–1865. 10.1136/ard.2011.150219 21798884

[B20] Bondeson J. Blom A. B. Wainwright S. Hughes C. Caterson B. van den Berg W. B. (2010). The role of synovial macrophages and macrophage-produced mediators in driving inflammatory and destructive responses in osteoarthritis. *Arthritis Rheum.* 62 647–657. 10.1002/art.27290 20187160

[B21] Bowles A. C. Willman M. A. Perucca Orfei C. Agarwal A. Correa D. (2020). Signature quality attributes of CD146+ mesenchymal stem/stromal cells correlate to high therapeutic and secretory potency. *Stem Cells* [Epub ahead of print]. 10.1002/stem.3196 32379908

[B22] Bravo B. Guisasola M. C. Vaquero J. Tirado I. Gortazar A. R. Forriol F. (2019). Gene expression, protein profiling, and chemotactic activity of infrapatellar fat pad mesenchymal stem cells in pathologies of the knee joint. *J. Cell Physiol.* 234 18917–18927. 10.1002/jcp.28532 30912165

[B23] Brouwers H. von Hegedus J. Toes R. Kloppenburg M. Ioan-Facsinay A. (2015). Lipid mediators of inflammation in rheumatoid arthritis and osteoarthritis. *Best Pract. Res. Clin. Rheumatol.* 29 741–755. 10.1016/j.berh.2016.02.003 27107510

[B24] Brumovsky P. R. (2016). Dorsal root ganglion neurons and tyrosine hydroxylase–an intriguing association with implications for sensation and pain. *Pain* 157 314–320. 10.1097/j.pain.0000000000000381 26447702 PMC4727984

[B25] Buckley C. T. Kelly D. J. (2012). Expansion in the presence of FGF-2 enhances the functional development of cartilaginous tissues engineered using infrapatellar fat pad derived MSCs. *J. Mech. Behav. Biomed. Mater.* 11 102–111. 10.1016/j.jmbbm.2011.09.004 22658159

[B26] Buckley C. T. Vinardell T. Thorpe S. D. Haugh M. G. Jones E. McGonagle D. (2010). Functional properties of cartilaginous tissues engineered from infrapatellar fat pad-derived mesenchymal stem cells. *J. Biomech.* 43 920–926. 10.1016/j.jbiomech.2009.11.005 20005518

[B27] Cai J. Wu J. Wang J. Li Y. Hu X. Luo S. (2020). Extracellular vesicles derived from different sources of mesenchymal stem cells: therapeutic effects and translational potential. *Cell Biosci.* 10:69. 10.1186/s13578-020-00427-x PMC7245623 32483483

[B28] Caplan A. I. Correa D. (2011). The MSC: an injury drugstore. *Cell Stem Cell.* 9 11–15. 10.1016/j.stem.2011.06.008 21726829 PMC3144500

[B29] Caspar-Bauguil S. Cousin B. Galinier A. Segafredo C. Nibbelink M. Andre M. (2005). Adipose tissues as an ancestral immune organ: site-specific change in obesity. *FEBS Lett.* 579 3487–3492. 10.1016/j.febslet.2005.05.031 15953605

[B30] Clements K. M. Ball A. D. Jones H. B. Brinckmann S. Read S. J. Murray F. (2009). Cellular and histopathological changes in the infrapatellar fat pad in the monoiodoacetate model of osteoarthritis pain. *Osteoarthr. Cartilage* 17 805–812. 10.1016/j.joca.2008.11.002 19114312

[B31] Clockaerts S. Bastiaansen-Jenniskens Y. M. Feijt C. De Clerck L. Verhaar J. A. Zuurmond A. M. (2012). Cytokine production by infrapatellar fat pad can be stimulated by interleukin 1beta and inhibited by peroxisome proliferator activated receptor alpha agonist. *Ann. Rheum. Dis.* 71 1012–1018. 10.1136/annrheumdis-2011-200688 22307941

[B32] Clockaerts S. Bastiaansen-Jenniskens Y. M. Runhaar J. Van Osch G. J. Van Offel J. F. Verhaar J. A. (2010). The infrapatellar fat pad should be considered as an active osteoarthritic joint tissue: a narrative review. *Osteoarthr. Cartilage* 18 876–882. 10.1016/j.joca.2010.03.014 20417297

[B33] Coelho M. Oliveira T. Fernandes R. (2013). Biochemistry of adipose tissue: an endocrine organ. *Arch. Med. Sci.* 9 191–200. 10.5114/aoms.2013.33181 23671428 PMC3648822

[B34] Cooper A. M. Khader S. A. (2007). IL-12p40: an inherently agonistic cytokine. *Trends Immunol.* 28 33–38. 10.1016/j.it.2006.11.002 17126601

[B35] Correa D. Somoza R. A. Lin P. Greenberg S. Rom E. Duesler L. (2015). Sequential exposure to fibroblast growth factors (FGF) 2, 9 and 18 enhances hMSC chondrogenic differentiation. *Osteoarthr. Cartilage* 23 443–453. 10.1016/j.joca.2014.11.013 25464167 PMC4692467

[B36] Cowan S. M. Hart H. F. Warden S. J. Crossley K. M. (2015). Infrapatellar fat pad volume is greater in individuals with patellofemoral joint osteoarthritis and associated with pain. *Rheumatol. Int.* 35 1439–1442. 10.1007/s00296-015-3250-0 25782586

[B37] Crema M. D. Felson D. T. Roemer F. W. Niu J. Marra M. D. Zhang Y. (2013). Peripatellar synovitis: comparison between non-contrast-enhanced and contrast-enhanced MRI and association with pain. The MOST study. *Osteoarthr. Cartilage* 21 413–418. 10.1016/j.joca.2012.12.006 23277189 PMC3578385

[B38] Davies L. C. Rosas M. Smith P. J. Fraser D. J. Jones S. A. Taylor P. R. (2011). A quantifiable proliferative burst of tissue macrophages restores homeostatic macrophage populations after acute inflammation. *Eur. J. Immunol.* 41 2155–2164. 10.1002/eji.201141817 21710478

[B39] De Bari C. Dell’Accio F. Tylzanowski P. Luyten F. P. (2001). Multipotent mesenchymal stem cells from adult human synovial membrane. *Arthritis Rheum.* 44 1928–1942. 10.1002/1529-0131(200108)44:8<1928::aid-art331>3.0.co;2-p 11508446

[B40] de Boer T. N. van Spil W. E. Huisman A. M. Polak A. A. Bijlsma J. W. Lafeber F. P. (2012). Serum adipokines in osteoarthritis; comparison with controls and relationship with local parameters of synovial inflammation and cartilage damage. *Osteoarthr. Cartilage* 20 846–853. 10.1016/j.joca.2012.05.002 22595228

[B41] de Jong A. J. Klein-Wieringa I. R. Andersen S. N. Kwekkeboom J. C. Herb-van Toorn L. de Lange-Brokaar B. J. E. (2017). Lack of high BMI-related features in adipocytes and inflammatory cells in the infrapatellar fat pad (IFP). *Arthritis Res. Ther.* 19 186. 10.1186/s13075-017-1395-9 PMC5553811 28800775

[B42] de Lange-Brokaar B. J. Ioan-Facsinay A. van Osch G. J. Zuurmond A. M. Schoones J. Toes R. E. (2012). Synovial inflammation, immune cells and their cytokines in osteoarthritis: a review. *Osteoarthr. Cartilage* 20 1484–1499. 10.1016/j.joca.2012.08.027 22960092

[B43] Decker R. S. Um H. B. Dyment N. A. Cottingham N. Usami Y. Enomoto-Iwamoto M. (2017). Cell origin, volume and arrangement are drivers of articular cartilage formation, morphogenesis and response to injury in mouse limbs. *Dev. Biol.* 426 56–68. 10.1016/j.ydbio.2017.04.006 28438606 PMC6046638

[B44] Ding D. C. Wu K. C. Chou H. L. Hung W. T. Liu H. W. Chu T. Y. (2015). Human infrapatellar fat pad-derived stromal cells have more potent differentiation capacity than other mesenchymal cells and can be enhanced by hyaluronan. *Cell Transplant.* 24 1221–1232. 10.3727/096368914x681937 24853696

[B45] Djouad F. Bony C. Haupl T. Uze G. Lahlou N. Louis-Plence P. (2005). Transcriptional profiles discriminate bone marrow-derived and synovium-derived mesenchymal stem cells. *Arthritis Res. Ther.* 7 R1304–R1315. 16277684 10.1186/ar1827 PMC1297577

[B46] do Amaral R. Almeida H. V. Kelly D. J. O’Brien F. J. Kearney C. J. (2017). Infrapatellar fat pad stem cells: from developmental biology to cell therapy. *Stem Cells Int.* 2017:6843727. 10.1155/2017/6843727 PMC5606137 29018484

[B47] Dragoo J. L. Johnson C. McConnell J. (2012). Evaluation and treatment of disorders of the infrapatellar fat pad. *Sports Med.* 42 51–67. 10.2165/11595680-000000000-00000 22149697

[B48] Dragoo J. L. Samimi B. Zhu M. Hame S. L. Thomas B. J. Lieberman J. R. (2003). Tissue-engineered cartilage and bone using stem cells from human infrapatellar fat pads. *J. Bone Joint Surg. Br.* 85 740–747. 10.1302/0301-620x.85b5.13587 12892203

[B49] Dumond H. Presle N. Terlain B. Mainard D. Loeuille D. Netter P. (2003). Evidence for a key role of leptin in osteoarthritis. *Arthritis Rheum.* 48 3118–3129. 10.1002/art.11303 14613274

[B50] English A. Jones E. A. Corscadden D. Henshaw K. Chapman T. Emery P. (2007). A comparative assessment of cartilage and joint fat pad as a potential source of cells for autologous therapy development in knee osteoarthritis. *Rheumatology* 46 1676–1683. 10.1093/rheumatology/kem217 17901063

[B51] Eymard F. Pigenet A. Citadelle D. Flouzat-Lachaniette C. H. Poignard A. Benelli C. (2014). Induction of an inflammatory and prodegradative phenotype in autologous fibroblast-like synoviocytes by the infrapatellar fat pad from patients with knee osteoarthritis. *Arthritis Rheumatol.* 66 2165–2174. 10.1002/art.38657 24719336

[B52] Farrell E. Both S. K. Odorfer K. I. Koevoet W. Kops N. O’Brien F. J. (2011). In-vivo generation of bone via endochondral ossification by in-vitro chondrogenic priming of adult human and rat mesenchymal stem cells. *BMC Musculoskelet. Disord.* 12:31. 10.1186/1471-2474-12-31 21281488 PMC3045394

[B53] Farrell E. van der Jagt O. P. Koevoet W. Kops N. van Manen C. J. Hellingman C. A. (2009). Chondrogenic priming of human bone marrow stromal cells: a better route to bone repair? *Tissue Eng. Part C Methods* 15 285–295. 10.1089/ten.tec.2008.0297 19505182

[B54] Favero M. El-Hadi H. Belluzzi E. Granzotto M. Porzionato A. Sarasin G. (2017). Infrapatellar fat pad features in osteoarthritis: a histopathological and molecular study. *Rheumatology* 56 1784–1793. 10.1093/rheumatology/kex287 28957567

[B55] Felson D. T. Niu J. Neogi T. Goggins J. Nevitt M. C. Roemer F. (2016). Synovitis and the risk of knee osteoarthritis: the MOST Study. *Osteoarthr. Cartilage* 24 458–464. 10.1016/j.joca.2015.09.013 26432512 PMC4761323

[B56] Feng X. Li Z. Wei J. Feng Z. Wu W. Zhao Y. (2018). Injectable cartilaginous template transformed BMSCs into vascularized bone. *Sci. Rep.* 8:8244. 10.1038/s41598-018-26472-8 PMC5973938 29844536

[B57] Fernandes T. L. Gomoll A. H. Lattermann C. Hernandez A. J. Bueno D. F. Amano M. T. (2020). Macrophage: a potential target on cartilage regeneration. *Front. Immunol.* 11:111. 10.3389/fimmu.2020.00111 32117263 PMC7026000

[B58] Ferro T. Santhagunam A. Madeira C. Salgueiro J. B. da Silva C. L. Cabral J. M. S. (2019). Successful isolation and ex vivo expansion of human mesenchymal stem/stromal cells obtained from different synovial tissue-derived (biopsy) samples. *J. Cell Physiol.* 234 3973–3984. 10.1002/jcp.27202 30146686

[B59] Fontanella C. G. Belluzzi E. Rossato M. Olivotto E. Trisolino G. Ruggieri P. (2019). Quantitative MRI analysis of infrapatellar and suprapatellar fat pads in normal controls, moderate and end-stage osteoarthritis. *Ann. Anat.* 221 108–114. 10.1016/j.aanat.2018.09.007 30292837

[B60] Frank-Bertoncelj M. Trenkmann M. Klein K. Karouzakis E. Rehrauer H. Bratus A. (2017). Epigenetically-driven anatomical diversity of synovial fibroblasts guides joint-specific fibroblast functions. *Nat. Commun.* 8:14852. 10.1038/ncomms14852 PMC5376654 28332497

[B61] Freeman M. A. Wyke B. (1967). The innervation of the knee joint. An anatomical and histological study in the cat. *J. Anat.* 101(Pt 3), 505–532. 6051731 PMC1270929

[B62] Galipeau J. Krampera M. Barrett J. Dazzi F. Deans R. J. DeBruijn J. (2016). International society for cellular therapy perspective on immune functional assays for mesenchymal stromal cells as potency release criterion for advanced phase clinical trials. *Cytotherapy* 18 151–159. 10.1016/j.jcyt.2015.11.008 26724220 PMC4745114

[B63] Gallagher J. Tierney P. Murray P. O’Brien M. (2005). The infrapatellar fat pad: anatomy and clinical correlations. *Knee Surg. Sports Traumatol. Arthrosc.* 13 268–272. 10.1007/s00167-004-0592-7 15678298

[B64] Garcia J. Mennan C. McCarthy H. S. Roberts S. Richardson J. B. Wright K. T. (2016a). Chondrogenic potency analyses of donor-matched chondrocytes and mesenchymal stem cells derived from bone marrow, infrapatellar fat pad, and subcutaneous fat. *Stem Cells Int.* 2016:6969726. 10.1155/2016/6969726 PMC5066011 27781068

[B65] Garcia J. Wright K. Roberts S. Kuiper J. H. Mangham C. Richardson J. (2016b). Characterisation of synovial fluid and infrapatellar fat pad derived mesenchymal stromal cells: the influence of tissue source and inflammatory stimulus. *Sci. Rep.* 6:24295. 10.1038/srep24295 PMC4829842 27073003

[B66] Garza J. R. Campbell R. E. Tjoumakaris F. P. Freedman K. B. Miller L. S. Santa Maria D. (2020). Clinical efficacy of intra-articular mesenchymal stromal cells for the treatment of knee osteoarthritis: a double-blinded prospective randomized controlled clinical trial. *Am. J. Sports Med.* 48 588–598. 10.1177/0363546519899923 32109160

[B67] Gentek R. Molawi K. Sieweke M. H. (2014). Tissue macrophage identity and self-renewal. *Immunol. Rev.* 262 56–73. 10.1111/imr.12224 25319327

[B68] Gierman L. M. Wopereis S. van El B. Verheij E. R. Werff-van, der Vat B. J. (2013). Metabolic profiling reveals differences in concentrations of oxylipins and fatty acids secreted by the infrapatellar fat pad of donors with end-stage osteoarthritis and normal donors. *Arthritis Rheum.* 65 2606–2614. 23839996 10.1002/art.38081

[B69] Ginhoux F. Guilliams M. (2016). Tissue-resident macrophage ontogeny and homeostasis. *Immunity* 44 439–449. 10.1016/j.immuni.2016.02.024 26982352

[B70] Ginhoux F. Jung S. (2014). Monocytes and macrophages: developmental pathways and tissue homeostasis. *Nat. Rev. Immunol.* 14 392–404. 10.1038/nri3671 24854589

[B71] Gomez-Aristizabal A. Gandhi R. Mahomed N. N. Marshall K. W. Viswanathan S. (2019). Synovial fluid monocyte/macrophage subsets and their correlation to patient-reported outcomes in osteoarthritic patients: a cohort study. *Arthritis Res. Ther.* 21:26. 10.1186/s13075-018-1798-2 PMC6339358 30658702

[B72] Hagmann S. Gotterbarm T. Muller T. Baesig A. M. Gantz S. Dreher T. (2013). The influence of bone marrow- and synovium-derived mesenchymal stromal cells from osteoarthritis patients on regulatory T cells in co-culture. *Clin. Exp. Immunol.* 173 454–462. 10.1111/cei.12122 23607395 PMC3949633

[B73] Han W. Aitken D. Zhu Z. Halliday A. Wang X. Antony B. (2016). Signal intensity alteration in the infrapatellar fat pad at baseline for the prediction of knee symptoms and structure in older adults: a cohort study. *Ann. Rheum. Dis.* 75 1783–1788. 10.1136/annrheumdis-2015-208360 26612337

[B74] Harrell C. R. Markovic B. S. Fellabaum C. Arsenijevic A. Volarevic V. (2019). Mesenchymal stem cell-based therapy of osteoarthritis: current knowledge and future perspectives. *Biomed. Pharmacother.* 109 2318–2326. 10.1016/j.biopha.2018.11.099 30551490

[B75] Heilmeier U. Mamoto K. Amano K. Eck B. Tanaka M. Bullen J. A. (2019). Infrapatellar fat pad abnormalities are associated with a higher inflammatory synovial fluid cytokine profile in young adults following ACL tear. *Osteoarthr. Cartilage* 28 82–91. 10.1016/j.joca.2019.09.001 31526878 PMC6935420

[B76] Hermida-Gomez T. Fuentes-Boquete I. Gimeno-Longas M. J. Muinos-Lopez E. Diaz-Prado S. de Toro F. J. (2011). Quantification of cells expressing mesenchymal stem cell markers in healthy and osteoarthritic synovial membranes. *J. Rheumatol.* 38 339–349. 10.3899/jrheum.100614 21078714

[B77] Hill C. L. Hunter D. J. Niu J. Clancy M. Guermazi A. Genant H. (2007). Synovitis detected on magnetic resonance imaging and its relation to pain and cartilage loss in knee osteoarthritis. *Ann. Rheum. Dis.* 66 1599–1603. 10.1136/ard.2006.067470 17491096 PMC2095318

[B78] Hindle P. Khan N. Biant L. Peault B. (2017). The infrapatellar fat pad as a source of perivascular stem cells with increased chondrogenic potential for regenerative medicine. *Stem Cells Transl. Med.* 6 77–87. 10.5966/sctm.2016-0040 28170170 PMC5442731

[B79] Hu P. Yang Q. Wang Q. Shi C. Wang D. Armato U. (2019). Mesenchymal stromal cells-exosomes: a promising cell-free therapeutic tool for wound healing and cutaneous regeneration. *Burns Trauma* 7:38. 10.1186/s41038-019-0178-8 PMC6933895 31890717

[B80] Ioan-Facsinay A. Kloppenburg M. (2013). An emerging player in knee osteoarthritis: the infrapatellar fat pad. *Arthritis Res. Ther.* 15:225. 10.1186/ar4422 24367915 PMC3979009

[B81] Ioan-Facsinay A. Kloppenburg M. (2017). Osteoarthritis: inflammation and fibrosis in adipose tissue of osteoarthritic joints. *Nat. Rev. Rheumatol.* 13 325–326. 10.1038/nrrheum.2017.53 28405000

[B82] Ioan-Facsinay A. Kwekkeboom J. C. Westhoff S. Giera M. Rombouts Y. van Harmelen V. (2013). Adipocyte-derived lipids modulate CD4+ T-cell function. *Eur. J. Immunol.* 43 1578–1587. 10.1002/eji.201243096 23504601

[B83] Ji Q. Zheng Y. Zhang G. Hu Y. Fan X. Hou Y. (2019). Single-cell RNA-seq analysis reveals the progression of human osteoarthritis. *Ann. Rheum. Dis.* 78 100–110. 10.1136/annrheumdis-2017-212863 30026257 PMC6317448

[B84] Jiang L. F. Fang J. H. Wu L. D. (2019). Role of infrapatellar fat pad in pathological process of knee osteoarthritis: future applications in treatment. *World J. Clin. Cases* 7 2134–2142. 10.12998/wjcc.v7.i16.2134 31531309 PMC6718789

[B85] Jiang T. Wang Z. Sun J. (2020). Human bone marrow mesenchymal stem cell-derived exosomes stimulate cutaneous wound healing mediates through TGF-beta/Smad signaling pathway. *Stem Cell Res. Ther.* 11:198. 10.1186/s13287-020-01723-6 PMC7245763 32448395

[B86] Jurgens W. J. van Dijk A. Doulabi B. Z. Niessen F. B. Ritt M. J. van Milligen F. J. (2009). Freshly isolated stromal cells from the infrapatellar fat pad are suitable for a one-step surgical procedure to regenerate cartilage tissue. *Cytotherapy* 11 1052–1064. 10.3109/14653240903219122 19929469

[B87] Kalaitzoglou E. Griffin T. M. Humphrey M. B. (2017). Innate immune responses and osteoarthritis. *Curr. Rheumatol. Rep.* 19:45. 10.1007/s11926-017-0672-6 28718060

[B88] Kandahari A. M. Yang X. Dighe A. S. Pan D. Cui Q. (2015). Recognition of immune response for the early diagnosis and treatment of osteoarthritis. *J. Immunol. Res.* 2015:192415. 10.1155/2015/192415 PMC4433702 26064995

[B89] Karnieli O. Friedner O. M. Allickson J. G. Zhang N. Jung S. Fiorentini D. (2017). A consensus introduction to serum replacements and serum-free media for cellular therapies. *Cytotherapy* 19 155–169. 10.1016/j.jcyt.2016.11.011 28017599

[B90] Karystinou A. Dell’Accio F. Kurth T. B. Wackerhage H. Khan I. M. Archer C. W. (2009). Distinct mesenchymal progenitor cell subsets in the adult human synovium. *Rheumatology* 48 1057–1064. 10.1093/rheumatology/kep192 19605375

[B91] Kennedy J. C. Alexander I. J. Hayes K. C. (1982). Nerve supply of the human knee and its functional importance. *Am. J. Sports Med.* 10 329–335. 10.1177/036354658201000601 6897495

[B92] Khan W. S. Tew S. R. Adesida A. B. Hardingham T. E. (2008). Human infrapatellar fat pad-derived stem cells express the pericyte marker 3G5 and show enhanced chondrogenesis after expansion in fibroblast growth factor-2. *Arthritis Res. Ther.* 10:R74. 10.1186/ar2448 PMC2575620 18598346

[B93] King L. K. March L. Anandacoomarasamy A. (2013). Obesity & osteoarthritis. *Indian J. Med. Res.* 138 185–193. 24056594 PMC3788203

[B94] Klein-Wieringa I. R. Andersen S. N. Kwekkeboom J. C. Giera M. de Lange-Brokaar B. J. van Osch G. J. (2013). Adipocytes modulate the phenotype of human macrophages through secreted lipids. *J. Immunol.* 191 1356–1363. 10.4049/jimmunol.1203074 23817431

[B95] Klein-Wieringa I. R. de Lange-Brokaar B. J. Yusuf E. Andersen S. N. Kwekkeboom J. C. Kroon H. M. (2016). Inflammatory cells in patients with endstage knee osteoarthritis: a comparison between the synovium and the infrapatellar fat pad. *J. Rheumatol.* 43 771–778. 10.3899/jrheum.151068 26980579

[B96] Klein-Wieringa I. R. Kloppenburg M. Bastiaansen-Jenniskens Y. M. Yusuf E. Kwekkeboom J. C. El-Bannoudi H. (2011). The infrapatellar fat pad of patients with osteoarthritis has an inflammatory phenotype. *Ann. Rheum. Dis.* 70 851–857. 10.1136/ard.2010.140046 21242232

[B97] Koh Y. G. Choi Y. J. (2012). Infrapatellar fat pad-derived mesenchymal stem cell therapy for knee osteoarthritis. *Knee* 19 902–907. 10.1016/j.knee.2012.04.001 22583627

[B98] Koh Y. G. Jo S. B. Kwon O. R. Suh D. S. Lee S. W. Park S. H. (2013). Mesenchymal stem cell injections improve symptoms of knee osteoarthritis. *Arthroscopy* 29 748–755. 10.1016/j.arthro.2012.11.017 23375182

[B99] Kohn D. Deiler S. Rudert M. (1995). Arterial blood supply of the infrapatellar fat pad. Anatomy and clinical consequences. *Arch. Orthop. Trauma Surg.* 114 72–75. 10.1007/bf00422828 7734236

[B100] Koizumi K. Ebina K. Hart D. A. Hirao M. Noguchi T. Sugita N. (2016). Synovial mesenchymal stem cells from osteo- or rheumatoid arthritis joints exhibit good potential for cartilage repair using a scaffold-free tissue engineering approach. *Osteoarthr. Cartilage* 24 1413–1422. 10.1016/j.joca.2016.03.006 26973329

[B101] Kordelas L. Rebmann V. Ludwig A. K. Radtke S. Ruesing J. Doeppner T. R. (2014). MSC-derived exosomes: a novel tool to treat therapy-refractory graft-versus-host disease. *Leukemia* 28 970–973. 10.1038/leu.2014.41 24445866

[B102] Kouroupis D. Bowles A. C. Willman M. A. Perucca Orfei C. Colombini A. Best T. M. (2019a). Infrapatellar fat pad-derived MSC response to inflammation and fibrosis induces an immunomodulatory phenotype involving CD10-mediated Substance P degradation. *Sci. Rep.* 9:10864. 10.1038/s41598-019-47391-2 PMC6659713 31350444

[B103] Kouroupis D. Sanjurjo-Rodriguez C. Jones E. Correa D. (2019b). Mesenchymal stem cell functionalization for enhanced therapeutic applications. *Tissue Eng. Part B Rev.* 25 55–77. 10.1089/ten.teb.2018.0118 30165783

[B104] Kouroupis D. B. A. Best T. M. Kaplan L. D. Correa D. (2020). CD10/neprilysin enrichment in infrapatellar fat pad-derived MSC under regulatory-compliant conditions: implications for efficient synovitis and fat pad fibrosis reversal. *Am. J. Sports Med.* 48 2013–2027. 10.1177/0363546520917699 32427493

[B105] Kriegova E. Manukyan G. Mikulkova Z. Gabcova G. Kudelka M. Gajdos P. (2018). Gender-related differences observed among immune cells in synovial fluid in knee osteoarthritis. *Osteoarthr. Cartilage* 26 1247–1256. 10.1016/j.joca.2018.04.016 29753948

[B106] Kubosch E. J. Lang G. Furst D. Kubosch D. Izadpanah K. Rolauffs B. (2018). The potential for synovium-derived stem cells in cartilage repair. *Curr. Stem Cell Res. Ther.* 13 174–184. 10.2174/1574888x12666171002111026 28969580

[B107] Kuptniratsaikul V. Thanakhumtorn S. Chinswangwatanakul P. Wattanamongkonsil L. Thamlikitkul V. (2009). Efficacy and safety of *Curcuma domestica* extracts in patients with knee osteoarthritis. *J. Altern. Complement. Med.* 15 891–897. 10.1089/acm.2008.0186 19678780

[B108] Lee S. Y. Nakagawa T. Reddi A. H. (2008). Induction of chondrogenesis and expression of superficial zone protein (SZP)/lubricin by mesenchymal progenitors in the infrapatellar fat pad of the knee joint treated with TGF-beta1 and BMP-7. *Biochem. Biophys. Res. Commun.* 376 148–153. 10.1016/j.bbrc.2008.08.138 18774772

[B109] Li F. Tang Y. Song B. Yu M. Li Q. Zhang C. (2019). Nomenclature clarification: synovial fibroblasts and synovial mesenchymal stem cells. *Stem Cell Res. Ther.* 10:260. 10.1186/s13287-019-1359-x PMC6701095 31426847

[B110] Li Y. S. Luo W. Zhu S. A. Lei G. H. (2017). T Cells in osteoarthritis: alterations and beyond. *Front. Immunol.* 8:356. 10.3389/fimmu.2017.00356 28424692 PMC5371609

[B111] Lieberthal J. Sambamurthy N. Scanzello C. R. (2015). Inflammation in joint injury and post-traumatic osteoarthritis. *Osteoarthr. Cartilage* 23 1825–1834. 10.1016/j.joca.2015.08.015 26521728 PMC4630675

[B112] Lim J. E. Chung E. Son Y. (2017). A neuropeptide, Substance-P, directly induces tissue-repairing M2 like macrophages by activating the PI3K/Akt/mTOR pathway even in the presence of IFNgamma. *Sci. Rep.* 7:9417. 10.1038/s41598-017-09639-7 PMC5573373 28842601

[B113] Liu Y. Buckley C. T. Almeida H. V. Mulhall K. J. Kelly D. J. (2014). Infrapatellar fat pad-derived stem cells maintain their chondrogenic capacity in disease and can be used to engineer cartilaginous grafts of clinically relevant dimensions. *Tissue Eng. Part A* 20 3050–3062. 10.1089/ten.tea.2014.0035 24785365 PMC4229863

[B114] Liu Y. Buckley C. T. Downey R. Mulhall K. J. Kelly D. J. (2012). The role of environmental factors in regulating the development of cartilaginous grafts engineered using osteoarthritic human infrapatellar fat pad-derived stem cells. *Tissue Eng. Part A* 18 1531–1541. 10.1089/ten.tea.2011.0575 22443147 PMC3419852

[B115] Loeuille D. Sauliere N. Champigneulle J. Rat A. C. Blum A. Chary-Valckenaere I. (2011). Comparing non-enhanced and enhanced sequences in the assessment of effusion and synovitis in knee OA: associations with clinical, macroscopic and microscopic features. *Osteoarthr. Cartilage* 19 1433–1439. 10.1016/j.joca.2011.08.010 21930225

[B116] Losina E. Walensky R. P. Reichmann W. M. Holt H. L. Gerlovin H. Solomon D. H. (2011). Impact of obesity and knee osteoarthritis on morbidity and mortality in older Americans. *Ann. Intern. Med.* 154 217–226. 21320937 10.1059/0003-4819-154-4-201102150-00001 PMC3260464

[B117] Macchi V. Stocco E. Stecco C. Belluzzi E. Favero M. Porzionato A. (2018). The infrapatellar fat pad and the synovial membrane: an anatomo-functional unit. *J. Anat.* 233 146–154. 10.1111/joa.12820 29761471 PMC6036933

[B118] Maekawa K. Furukawa H. Kanazawa Y. Hijioka A. Suzuki K. Fujimoto S. (1996). Electron and immunoelectron microscopy on healing process of the rat anterior cruciate ligament after partial transection: the roles of multipotent fibroblasts in the synovial tissue. *Histol. Histopathol.* 11 607–619. 8839751

[B119] Marsano A. Millward-Sadler S. J. Salter D. M. Adesida A. Hardingham T. Tognana E. (2007). Differential cartilaginous tissue formation by human synovial membrane, fat pad, meniscus cells and articular chondrocytes. *Osteoarthr. Cartilage* 15 48–58. 10.1016/j.joca.2006.06.009 16891129

[B120] Mashaghi A. Marmalidou A. Tehrani M. Grace P. M. Pothoulakis C. Dana R. (2016). Neuropeptide substance P and the immune response. *Cell Mol. Life Sci.* 73 4249–4264. 10.1007/s00018-016-2293-z 27314883 PMC5056132

[B121] Mata-Essayag S. Magaldi S. de Capriles C. H. Henao L. Garrido L. Pacillo V. (2001). Mucor indicus necrotizing fasciitis. *Int. J. Dermatol.* 40 406–408. 10.1046/j.1365-4362.2001.01246-3.x 11589747

[B122] Matarese G. Leiter E. H. La Cava A. (2007). Leptin in autoimmunity: many questions, some answers. *Tissue Antigens* 70 87–95. 10.1111/j.1399-0039.2007.00886.x 17610413

[B123] Matas J. Orrego M. Amenabar D. Infante C. Tapia-Limonchi R. Cadiz M. I. (2019). Umbilical cord-derived mesenchymal stromal cells (MSCs) for knee osteoarthritis: repeated MSC dosing is superior to a single msc dose and to hyaluronic acid in a controlled randomized phase I/II Trial. *Stem Cells Transl. Med.* 8 215–224. 10.1002/sctm.18-0053 30592390 PMC6392367

[B124] Mathiessen A. Conaghan P. G. (2017). Synovitis in osteoarthritis: current understanding with therapeutic implications. *Arthritis Res. Ther.* 19 18. 10.1186/s13075-017-1229-9 PMC5289060 28148295

[B125] Mathis D. (2013). Immunological goings-on in visceral adipose tissue. *Cell Metab.* 17 851–859. 10.1016/j.cmet.2013.05.008 23747244 PMC4264591

[B126] McIntyre J. A. Jones I. A. Han B. Vangsness C. T. Jr. (2018). Intra-articular mesenchymal stem cell therapy for the human joint: a systematic review. *Am. J. Sports Med.* 46 3550–3563. 10.1177/0363546517735844 29099618

[B127] Mendicino M. Bailey A. M. Wonnacott K. Puri R. K. Bauer S. R. (2014). MSC-based product characterization for clinical trials: an FDA perspective. *Cell Stem Cell.* 14 141–145. 10.1016/j.stem.2014.01.013 24506881

[B128] Meng Q. Qiu B. (2020). Exosomal MicroRNA-320a derived from mesenchymal stem cells regulates rheumatoid arthritis fibroblast-like synoviocyte activation by suppressing CXCL9 expression. *Front. Physiol.* 11:441. 10.3389/fphys.2020.00441 32528301 PMC7264418

[B129] Mesallati T. Buckley C. T. Kelly D. J. (2017). Engineering cartilaginous grafts using chondrocyte-laden hydrogels supported by a superficial layer of stem cells. *J. Tissue Eng. Regen. Med.* 11 1343–1353. 10.1002/term.2033 26010516

[B130] Mesallati T. Sheehy E. J. Vinardell T. Buckley C. T. Kelly D. J. (2015). Tissue engineering scaled-up, anatomically shaped osteochondral constructs for joint resurfacing. *Eur. Cell Mater.* 30 163–85; discussion85–86. 26412388 10.22203/ecm.v030a12

[B131] Mizuno H. Tobita M. Uysal A. C. (2012). Concise review: adipose-derived stem cells as a novel tool for future regenerative medicine. *Stem Cells* 30 804–810. 10.1002/stem.1076 22415904

[B132] Mizuno M. Katano H. Mabuchi Y. Ogata Y. Ichinose S. Fujii S. (2018). Specific markers and properties of synovial mesenchymal stem cells in the surface, stromal, and perivascular regions. *Stem Cell Res. Ther.* 9:123. 10.1186/s13287-018-0870-9 PMC5930798 29720268

[B133] Mochizuki T. Muneta T. Sakaguchi Y. Nimura A. Yokoyama A. Koga H. (2006). Higher chondrogenic potential of fibrous synovium- and adipose synovium-derived cells compared with subcutaneous fat-derived cells: distinguishing properties of mesenchymal stem cells in humans. *Arthritis Rheum.* 54 843–853. 10.1002/art.21651 16508965

[B134] Mustonen A. M. Kakela R. Lehenkari P. Huhtakangas J. Turunen S. Joukainen A. (2019). Distinct fatty acid signatures in infrapatellar fat pad and synovial fluid of patients with osteoarthritis versus rheumatoid arthritis. *Arthritis Res. Ther.* 21:124. 10.3109/08916934.2015.1113267 31118103 PMC6532171

[B135] Muttigi M. S. Kim B. J. Choi B. Yoshie A. Kumar H. Han I. (2018). Matrilin-3 codelivery with adipose-derived mesenchymal stem cells promotes articular cartilage regeneration in a rat osteochondral defect model. *J. Tissue Eng. Regen. Med.* 12 667–675. 10.1002/term.2485 28556569

[B136] Nair A. Kanda V. Bush-Joseph C. Verma N. Chubinskaya S. Mikecz K. (2012). Synovial fluid from patients with early osteoarthritis modulates fibroblast-like synoviocyte responses to toll-like receptor 4 and toll-like receptor 2 ligands via soluble CD14. *Arthritis Rheum.* 64 2268–2277. 10.1002/art.34495 22492243 PMC3386375

[B137] Nimura A. Muneta T. Koga H. Mochizuki T. Suzuki K. Makino H. (2008). Increased proliferation of human synovial mesenchymal stem cells with autologous human serum: comparisons with bone marrow mesenchymal stem cells and with fetal bovine serum. *Arthritis Rheum.* 58 501–510. 10.1002/art.23219 18240254

[B138] O’HEireamhoin S. Buckley C. T. Jones E. McGonagle D. Mulhall K. J. Kelly D. J. (2013). Recapitulating aspects of the oxygen and substrate environment of the damaged joint milieu for stem cell-based cartilage tissue engineering. *Tissue Eng. Part C Methods* 19 117–127. 10.1089/ten.tec.2012.0142 22834895

[B139] Orozco L. Munar A. Soler R. Alberca M. Soler F. Huguet M. (2014). Treatment of knee osteoarthritis with autologous mesenchymal stem cells: two-year follow-up results. *Transplantation* 97 e66–e68. 10.1097/tp.0000000000000167 24887752

[B140] Orr C. Vieira-Sousa E. Boyle D. L. Buch M. H. Buckley C. D. Canete J. D. (2017). Synovial tissue research: a state-of-the-art review. *Nat. Rev. Rheumatol.* 13:630. 10.1038/nrrheum.2017.161 28935945

[B141] Ospelt C. (2017). Synovial fibroblasts in. *RMD Open* 3:e000471. 10.1136/rmdopen-2017-000471 29081987 PMC5652455

[B142] Pachler K. Lener T. Streif D. Dunai Z. A. Desgeorges A. Feichtner M. (2017). A good manufacturing practice-grade standard protocol for exclusively human mesenchymal stromal cell-derived extracellular vesicles. *Cytotherapy* 19 458–472. 10.1016/j.jcyt.2017.01.001 28188071

[B143] Pan F. Han W. Wang X. Liu Z. Jin X. Antony B. (2015). A longitudinal study of the association between infrapatellar fat pad maximal area and changes in knee symptoms and structure in older adults. *Ann. Rheum. Dis.* 74 1818–1824. 10.1136/annrheumdis-2013-205108 24833783

[B144] Paul F. Arkin Y. Giladi A. Jaitin D. A. Kenigsberg E. Keren-Shaul H. (2015). Transcriptional heterogeneity and lineage commitment in myeloid progenitors. *Cell* 163 1663–1677. 10.1016/j.cell.2015.11.013 26627738

[B145] Pelletier J. P. Martel-Pelletier J. Abramson S. B. (2001). Osteoarthritis, an inflammatory disease: potential implication for the selection of new therapeutic targets. *Arthritis Rheum.* 44 1237–1247. 10.1002/1529-0131(200106)44:6<1237::aid-art214>3.0.co;2-f 11407681

[B146] Prabhakar A. Lynch A. P. Ahearne M. (2016). Self-assembled infrapatellar fat-pad progenitor cells on a poly-epsilon-caprolactone film for cartilage regeneration. *Artif. Organs* 40 376–384. 10.1111/aor.12565 26516689

[B147] Regev A. Teichmann S. A. Lander E. S. Amit I. Benoist C. Birney E. (2017). The human cell atlas. *eLife* 6:e27041. 10.7554/eLife.27041 PMC5762154 29206104

[B148] Roelofs A. J. Zupan J. Riemen A. H. K. Kania K. Ansboro S. White N. (2017). Joint morphogenetic cells in the adult mammalian synovium. *Nat. Commun.* 8:15040. 10.1038/ncomms15040 PMC5493527 28508891

[B149] Roemer F. W. Guermazi A. Zhang Y. Yang M. Hunter D. J. Crema M. D. (2009). Hoffa’s fat pad: evaluation on unenhanced mr images as a measure of patellofemoral synovitis in osteoarthritis. *Am. J. Roentgenol.* 192 1696–1700. 10.2214/ajr.08.2038 19457837

[B150] Rohde E. Pachler K. Gimona M. (2019). Manufacturing and characterization of extracellular vesicles from umbilical cord-derived mesenchymal stromal cells for clinical testing. *Cytotherapy* 21 581–592. 10.1016/j.jcyt.2018.12.006 30979664

[B151] Sakaguchi Y. Sekiya I. Yagishita K. Muneta T. (2005). Comparison of human stem cells derived from various mesenchymal tissues: superiority of synovium as a cell source. *Arthritis Rheum.* 52 2521–2529. 10.1002/art.21212 16052568

[B152] Scanzello C. R. Goldring S. R. (2012). The role of synovitis in osteoarthritis pathogenesis. *Bone* 51 249–257. 10.1016/j.bone.2012.02.012 22387238 PMC3372675

[B153] Schnoor M. Alcaide P. Voisin M. B. van Buul J. D. (2016). Recruitment of immune cells into inflamed tissues: consequences for endothelial barrier integrity and tissue functionality. *Mediat. Inflamm.* 2016:1561368. 10.1155/2016/1561368 PMC4773555 26989330

[B154] Scotti C. Piccinini E. Takizawa H. Todorov A. Bourgine P. Papadimitropoulos A. (2013). Engineering of a functional bone organ through endochondral ossification. *Proc. Natl. Acad. Sci. U.S.A.* 110 3997–4002. 10.1073/pnas.1220108110 23401508 PMC3593845

[B155] Sekiya I. Muneta T. Horie M. Koga H. (2015). Arthroscopic transplantation of synovial stem cells improves clinical outcomes in knees with cartilage defects. *Clin. Orthop. Relat. Res.* 473 2316–2326. 10.1007/s11999-015-4324-8 25925939 PMC4457765

[B156] Shimomura K. Yasui Y. Koizumi K. Chijimatsu R. Hart D. A. Yonetani Y. (2018). First-in-human pilot study of implantation of a scaffold-free tissue-engineered construct generated from autologous synovial mesenchymal stem cells for repair of knee chondral lesions. *Am. J. Sports Med.* 46 2384–2393. 10.1177/0363546518781825 29969043

[B157] Siciliano C. Bordin A. Ibrahim M. Chimenti I. Cassiano F. Gatto I. (2016). The adipose tissue of origin influences the biological potential of human adipose stromal cells isolated from mediastinal and subcutaneous fat depots. *Stem Cell Res.* 17 342–351. 10.1016/j.scr.2016.07.010 27614132

[B158] Sivasubramaniyan K. Koevoet W. Hakimiyan A. A. Sande M. Farrell E. Hoogduijn M. J. (2019). Cell-surface markers identify tissue resident multipotential stem/stromal cell subsets in synovial intimal and sub-intimal compartments with distinct chondrogenic properties. *Osteoarthr. Cartilage* 27 1831–1840. 10.1016/j.joca.2019.08.006 31536814

[B159] Sokolove J. Lepus C. M. (2013). Role of inflammation in the pathogenesis of osteoarthritis: latest findings and interpretations. *Ther. Adv. Musculoskelet. Dis.* 5 77–94. 10.1177/1759720x12467868 23641259 PMC3638313

[B160] Soler R. Orozco L. Munar A. Huguet M. Lopez R. Vives J. (2016). Final results of a phase I-II trial using ex vivo expanded autologous Mesenchymal Stromal Cells for the treatment of osteoarthritis of the knee confirming safety and suggesting cartilage regeneration. *Knee* 23 647–654. 10.1016/j.knee.2015.08.013 26783191

[B161] Spitsin S. Meshki J. Winters A. Tuluc F. Benton T. D. Douglas S. D. (2017). Substance P-mediated chemokine production promotes monocyte migration. *J. Leukoc. Biol.* 101 967–973. 10.1189/jlb.1ab0416-188rr 28366881 PMC5346177

[B162] Stagg J. Galipeau J. (2013). Mechanisms of immune modulation by mesenchymal stromal cells and clinical translation. *Curr. Mol. Med.* 13 856–867. 10.2174/1566524011313050016 23642066

[B163] Sun A. R. Panchal S. K. Friis T. Sekar S. Crawford R. Brown L. (2017). Obesity-associated metabolic syndrome spontaneously induces infiltration of pro-inflammatory macrophage in synovium and promotes osteoarthritis. *PLoS One* 12:e0183693. 10.1371/journal.pone.0183693 28859108 PMC5578643

[B164] Sun Y. Chen S. Pei M. (2018). Comparative advantages of infrapatellar fat pad: an emerging stem cell source for regenerative medicine. *Rheumatology* 57 2072–2086. 10.1093/rheumatology/kex487 29373763 PMC6256334

[B165] Suvas S. (2017). Role of substance P neuropeptide in inflammation, wound healing, and tissue homeostasis. *J. Immunol.* 199 1543–1552. 10.4049/jimmunol.1601751 28827386 PMC5657331

[B166] Svensson V. Vento-Tormo R. Teichmann S. A. (2018). Exponential scaling of single-cell RNA-seq in the past decade. *Nat. Protoc.* 13 599–604. 10.1038/nprot.2017.149 29494575

[B167] Tangchitphisut P. Srikaew N. Numhom S. Tangprasittipap A. Woratanarat P. Wongsak S. (2016). Infrapatellar fat pad: an alternative source of adipose-derived mesenchymal stem cells. *Arthritis* 2016:4019873. 10.1155/2016/4019873 PMC4861778 27239342

[B168] To K. Zhang B. Romain K. Mak C. Khan W. (2019). Synovium-derived mesenchymal stem cell transplantation in cartilage regeneration: a PRISMA review of in vivo studies. *Front. Bioeng. Biotechnol.* 7:314. 10.3389/fbioe.2019.00314 31803726 PMC6873960

[B169] Toghraie F. S. Chenari N. Gholipour M. A. Faghih Z. Torabinejad S. Dehghani S. (2011). Treatment of osteoarthritis with infrapatellar fat pad derived mesenchymal stem cells in Rabbit. *Knee* 18 71–75. 10.1016/j.knee.2010.03.001 20591677

[B170] Toussirot E. Streit G. Wendling D. (2007). The contribution of adipose tissue and adipokines to inflammation in joint diseases. *Curr. Med. Chem.* 14 1095–1100. 10.2174/092986707780362826 17456023

[B171] Tu J. Hong W. Guo Y. Zhang P. Fang Y. Wang X. (2019). Ontogeny of synovial macrophages and the roles of synovial macrophages from different origins in arthritis. *Front. Immunol.* 10:1146. 10.3389/fimmu.2019.01146 31231364 PMC6558408

[B172] Tu J. Hong W. Zhang P. Wang X. Korner H. Wei W. (2018). Ontology and function of fibroblast-like and macrophage-like synoviocytes: how do they talk to each other and can they be targeted for rheumatoid arthritis therapy? *Front. Immunol.* 9:1467. 10.3389/fimmu.2018.01467 29997624 PMC6028561

[B173] Uccelli A. de Rosbo N. K. (2015). The immunomodulatory function of mesenchymal stem cells: mode of action, and pathways. *Ann. N. Y. Acad. Sci.* 1351 114–126. 10.1111/nyas.12815 26152292

[B174] Vega A. Martin-Ferrero M. A. Del Canto F. Alberca M. Garcia V. Munar A. (2015). Treatment of knee osteoarthritis with allogeneic bone marrow mesenchymal stem cells: a randomized controlled trial. *Transplantation* 99 1681–1690. 10.1097/tp.0000000000000678 25822648

[B175] Vilalta M. Degano I. R. Bago J. Gould D. Santos M. Garcia-Arranz M. (2008). Biodistribution, long-term survival, and safety of human adipose tissue-derived mesenchymal stem cells transplanted in nude mice by high sensitivity non-invasive bioluminescence imaging. *Stem Cells Dev.* 17 993–1003. 18537463 10.1089/scd.2007.0201

[B176] Vinardell T. Buckley C. T. Thorpe S. D. Kelly D. J. (2011). Composition–function relations of cartilaginous tissues engineered from chondrocytes and mesenchymal stem cells isolated from bone marrow and infrapatellar fat pad. *J. Tissue Eng. Regen. Med.* 5 673–683. 10.1002/term.357 21953865

[B177] Vinardell T. Sheehy E. J. Buckley C. T. Kelly D. J. (2012). A comparison of the functionality and in vivo phenotypic stability of cartilaginous tissues engineered from different stem cell sources. *Tissue Eng. Part A* 18 1161–1170. 10.1089/ten.tea.2011.0544 22429262 PMC3360504

[B178] Wickham M. Q. Erickson G. R. Gimble J. M. Vail T. P. Guilak F. (2003). Multipotent stromal cells derived from the infrapatellar fat pad of the knee. *Clin. Orthop. Relat. Res.* 412 196–212. 10.1097/01.blo.0000072467.53786.ca 12838072

[B179] Witwer K. W. Van Balkom B. W. M. Bruno S. Choo A. Dominici M. Gimona M. (2019). Defining mesenchymal stromal cell (MSC)-derived small extracellular vesicles for therapeutic applications. *J. Extracell Vesicles* 8:1609206. 10.1080/20013078.2019.1609206 31069028 PMC6493293

[B180] Woodley S. J. Latimer C. P. Meikle G. R. Stringer M. D. (2012). Articularis genus: an anatomic and MRI study in cadavers. *J. Bone Joint Surg. Am.* 94 59–67. 10.2106/jbjs.k.00157 22218383

[B181] Wu C. L. Harasymowicz N. S. Klimak M. A. Collins K. H. Guilak F. (2020). The role of macrophages in osteoarthritis and cartilage repair. *Osteoarthr. Cartilage* 28 544–554. 10.1016/j.joca.2019.12.007 31926267 PMC7214213

[B182] Ye K. Felimban R. Traianedes K. Moulton S. E. Wallace G. G. Chung J. (2014). Chondrogenesis of infrapatellar fat pad derived adipose stem cells in 3D printed chitosan scaffold. *PLoS One* 9:e99410. 10.1371/journal.pone.0099410 24918443 PMC4053433

[B183] Yoshitomi H. (2019). Regulation of immune responses and chronic inflammation by fibroblast-like synoviocytes. *Front. Immunol.* 10:1395. 10.3389/fimmu.2019.01395 31275325 PMC6593115

[B184] Yusuf E. (2012). Metabolic factors in osteoarthritis: obese people do not walk on their hands. *Arthritis Res. Ther.* 14:123. 10.1186/ar3894 22809017 PMC3580548

[B185] Zeyda M. Farmer D. Todoric J. Aszmann O. Speiser M. Gyori G. (2007). Human adipose tissue macrophages are of an anti-inflammatory phenotype but capable of excessive pro-inflammatory mediator production. *Int. J. Obes.* 31 1420–1428. 10.1038/sj.ijo.0803632 17593905

[B186] Zhao Y. Zou W. Du J. Zhao Y. (2018). The origins and homeostasis of monocytes and tissue-resident macrophages in physiological situation. *J. Cell Physiol.* 233 6425–6439. 10.1002/jcp.26461 29323706

